# A mixed-integer slacks-based measure data envelopment analysis for efficiency measuring of German university hospitals

**DOI:** 10.1007/s10729-022-09620-5

**Published:** 2022-11-17

**Authors:** Mansour Zarrin

**Affiliations:** Faculty of Business and Economics, University of Augsburg, Universitätsstraße 16, 86159 Augsburg, Germany

**Keywords:** Data envelopment analysis, Integer-valued measures, Flexible measures, University hospitals, Productivity analysis

## Abstract

Standard Data Envelopment Analysis (DEA) models consider continuous-valued and known input and output statuses for measures. This paper proposes an extended Slacks-Based Measure (SBM) DEA model to accommodate flexible (a measure that can play the role of input and output) and integer measures simultaneously. A flexible measure’s most appropriate role (designation) is determined by maximizing the technical efficiency of each unit. The main advantage of the proposed model is that all inputs, outputs, and flexible measures can be expressed in integer values without inflation of efficiency scores since they are directly calculated by modifying input and output inefficiencies. Furthermore, we illustrate and examine the application of the proposed models with 28 university hospitals in Germany. We investigate the differences and common properties of the proposed models with the literature to shed light on both teaching and general inefficiencies. Results of inefficiency decomposition indicate that “Third-party funding income” that university hospitals receive from the research-granting agencies dominates the other inefficiencies sources. The study of the efficiency scores is then followed up with a second-stage regression analysis based on efficiency scores and environmental factors. The result of the regression analysis confirms the conclusion derived from the inefficiency decomposition analysis.

## Introduction

Data Envelopment Analysis (DEA) is a nonparametric approach introduced by Charnes, et al. [1] to estimate the relative efficiency of a set of homogeneous Decision-Making Units (DMUs) where utilize similar inputs to generate similar outputs. This basic model (from now referred to as CCR) has come up with a fruitful area for efficiency evaluation. DEA models can be categorized as radial and non-radial. The CCR represents the radial models where they cope with relative changes of input and/or output factors so that, the efficiency score imitates the proportional maximum output (input) expansion (reduction) rate which is common to all outputs (inputs). However, in many practical applications, not all inputs/outputs operate proportionally. Consider the hospitals as an instance, we utilize beds, physicians, and nurses as inputs where they may not change proportionally. There might be several non-radial slacks left that play an imperative role in reporting managerial efficiency, but they are not taken into account in the radial model. The Slacks-Based Measure (SBM) approach, on the other hand, disregards proportional changes and evaluates efficiency by considering the input excess and output shortfall (slacks) directly [2]. Both non-radial and radial DEA models have been well-documented from the theoretical perspective in the literature [3]. In addition to the theoretical development of DEA models, their application is significantly growing since it is well-known as a reliable methodology, e.g., for healthcare [4–8]. However, to our knowledge, most of the previous studies done in the field of teaching hospital performance assessment use the basic DEA models and pay no attention to two principal challenges that exist in the real-world situation: integer-valued amounts and flexible measures. In the following subsections, these two issues are adequately addressed.

### Integrality-constrained DEA

**Fig. 1:**
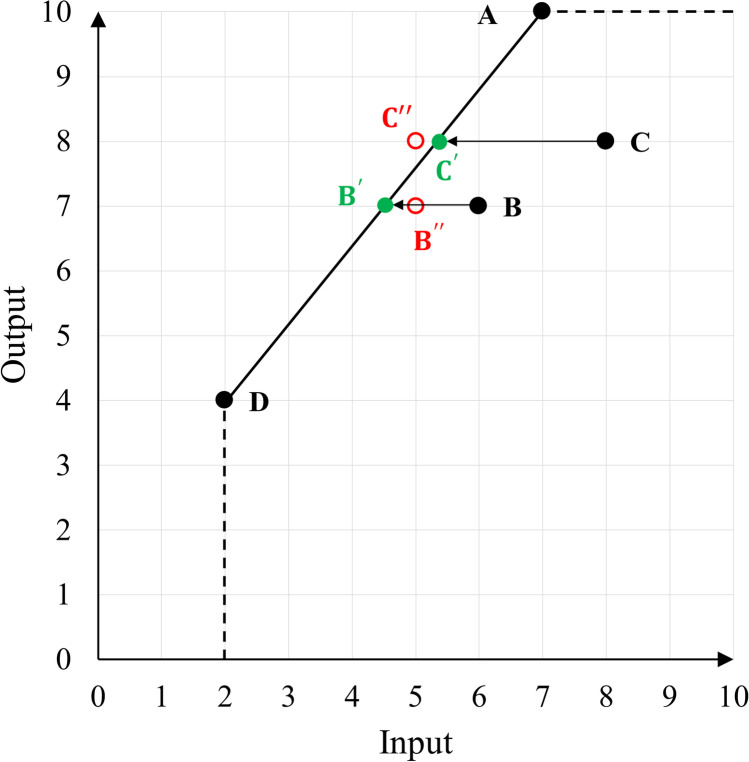
An example of infeasibility in the presence of integer-valued input under the VRS setting

Conventional DEA models consider that inputs and outputs are continuous values. However, we face many real situations in which one or some of the inputs/outputs are unavoidably integer values, for instance, the number of beds (as input) and outpatients (as output) in the hospital performance assessment. Usually the first step in DEA application, after identifying the list of inputs and outputs, is determining the suitable technology or the Production Possibility Set (PPS). These technologies are grouped as non-convex and convex. The non-convex Free Disposal Hull (FDH) [9] and the convex Constant and Variable Returns to Scale (CRS and VRS respectively) technologies are the most common choices. FDH targets are always feasible when some of the inputs/outputs are integer-valued since they project the units whose efficiency is to be evaluated onto one of the existing DMUs. In contrast, the PPS in both CRS and VRS assumes feasible operating points that are a convex combination of evaluating units without essentially considering any integrality constraint of some inputs/outputs. While imposing the integrality constraints by rounding off the optimum solution of the large integer values may have not a major effect on the optimality, it is not the case with small integer values where a few units less or more can make a significant difference in the optimality [10–12]. Assuming the integer-valued inputs/outputs as continuous values and arbitrarily rounding up (or down) of them may easily cause infeasibility (i.e., an operation point out of the PPS) or to a dominated (inferior) operating unit as mentioned by [13]. As illustrated by a single input single output example in Fig. 1, DMUs *B* and *C* are inefficient and their reference set includes DMUs *A* and *D*. The input excess of DMUs *B* and *C* are 1.5 and 2.67. That means 1.5 and 2.67 units reduction in the input of DMU *B* and *C*, respectively, project them on the green marks *B*^′^ and *C*^′^ on the efficient frontier (blue dashed line). However, if they are integer-valued, arbitrarily rounding up the input excess of *C* to 3.0 causes infeasibility, in other words, the red mark *C*^′′^ where is out of the PPS. As it is clear from the graph, an arbitrary rounding down the input excess of B to 1.0 (*B*^′′^) does not approach the efficient frontier.

### Flexible measure DEA

The usual setting for a DEA study is to evaluate DMUs, such as hospitals, according to specific input and output factors. The output represents the result of the DMU, while the input is intended to describe what led to the creation of that output. However, there exist some situations where some measures can play the role of either output or input. Consider, for example, the number of graduates or trainee nurses in a university hospital. These measures can constitute either input (two available human resources to the hospital) or output (trained staff, henceforth an advantage resulting from teaching/research funding). These measures are known as flexible or dual-role measures in DEA literature. In cases of ambiguity, it is imperative to adhere to the most equitable treatment possible for a particular DMU in order to decide the status of a variable. This ambiguity is further compounded if one views performance measurement from the perspective of an administrative organization as a manager. As with university hospitals, deciding whether graduates are to be regarded as an input or an output can have a tremendous impact on the funding received by each individual candidate. Therefore, these hospitals have a financial interest in using the least controversial and most fair method possible to assess efficiency.

The use of a factor as both an input and an output is not completely unheard of in the DEA framework. The status of flexible variables in DEA settings can be determined by at least three approaches. The first approach treats flexible measures on both the input and output sides simultaneously. For example, Beasley [14] treats “research funding” on both the input and output sides at the same time in university efficiency measuring. Later, Cook, et al. [15] show that this treatment is not completely appropriate. Second, and perhaps most obvious, is to consider the issue from the standpoint of individual DMUs. A DEA model is run specifically for each DMU to determine the optimal role of each flexible measure. It could then be decided based on the majority choice among the DMUs what the overall input versus output status of any flexible measure is. In this case, it would seem to be the least controversial way to choose to apply a simple majority decision rule [16]. As a third alternative, it is possible to consider the situation from the viewpoint of the manager of a collection of DMUs. Specifically, consider defining each flexible variable as an input or output so that the average or aggregate efficiency of the set of DMUs is maximized. An approach such as this would be useful if ties are encountered on a case-by-case basis [15–17].

This study aims to develop an SBM DEA model that includes integer- and continuous-valued inputs, outputs, and flexible measures at the same time. Each flexible measure in the proposed model can be viewed as input, output, or both. The flexible measure’s optimal role for the DMU being evaluated is dedicated to maximizing its technical efficiency. As a result, both the input surplus and output shortfall (slacks) may be present in the optimal solution set for each inefficient DMU. For efficient DMUs, flexible measures can be viewed both as input and output without affecting the degree of efficiency, since they are the ones with no slacks in their optimal solution. The proposed model has another advantage in that all three classes of measures can only take integer-valued slacks.

The rest of this paper is structured as follows. The literature on theoretical and application issues is reviewed in Section 2. In section 3, we review the advances in SBM DEA models in the presence of integer-valued and flexible measures. Then, we propose a new model as well as a new efficiency index. Section 4 presents the case study of the German university hospitals and the results of running the proposed models (efficiencies and slacks) and the developed ones in the literature. We also analyze the obtained results from the models and investigate the inefficiencies sources in this section. Finally, we wrap up our study and findings in Section 5.

## Literature review

This section provides an overview of the theoretical and application literature. We begin by examining studies related to the measurement of university hospital performance. Afterward, we review the theoretical development of the DEA models for integer-valued and flexible measures.

From a practical perspective, this study focuses on the performance evaluation of university hospitals. As reported in the health economics literature, teaching and university hospitals are more expensive than non-teaching counterparts (e.g., acute and general hospitals) since they engage in not only patient care but also in medical education and research. Therefore, this teaching/researching mission should be appropriately captured by defining proper measurements in the performance assessment process. One of the first studies in this field is conducted by Grosskopf, et al. [18]. They compare the patient service provision of both non-teaching and teaching hospitals by the means of the basic DEA model. They apply the DEA model to a dataset that includes 556 non-teaching and 236 teaching hospitals in the US. Their results specify around 10% of the teaching hospitals can efficiently compare with non-teaching counterparts. Later, Grosskopf, et al. [19] evaluate the relative scale and technical efficiencies of 254 US teaching hospitals. They find that intensified competition results in superior efficiency deprived of cooperating teaching intensity. Ozcan, et al. [20] evaluate the performance of Brazilian teaching hospitals considering both medical care and teaching/research. They conclude their study by indicating the required changes for the inefficient teaching hospitals as some recommendations for public financing and teaching ratios. In another study, Lobo, et al. [21] study the efficiency of 104 teaching hospitals in Brazil. They use a two-stage weighted DEA model followed by logistic regression analysis in the second stage to examine the effect of non-discretionary variables (e.g., ownership type) on the efficiency scores. The result of the regression shows no significant relationship between ownership and efficiency. In the case of the German hospital market, recently, Schneider, et al. [22] conduct a study on efficiency analysis of German hospitals (including both teaching and non-teaching) with a focus on investigating the relation between medical urgency and efficiency. Their results show a lower efficiency for teaching hospitals compared to the non-teaching ones. This is because the same set of input/output with the non-teaching hospitals is only used in their DEA model and the teaching function is not apprehended.

The integer DEA models have not attracted too much attention even though this situation can happen frequently in real-case applications. One reason for this may be the commitment of the DEA researchers to Linear Programming (LP) models since most LP DEA models can be proficiently solved even for big datasets using non-commercial solvers. To our knowledge, Lozanoand Villa [23] introduce the first DEA model whose inputs and outputs are intuitively constrained to take integer values only. They model their problem as a Mixed-Integer Linear Programming (MILP) for assessing efficiency of DMUs. Kuosmanenand Matin [11] develop a new axiomatic foundation (namely, “natural disposability” and “natural divisibility”) for DEA subject to the integrality constraints. They derive a new DEA PPS that fulfills the minimum extrapolation principle under their advanced axioms. They also present an MILP formula for assessing efficiency scores of Iranian university departments under the CRS assumption. Later, Kazemi Matinand Kuosmanen [24] extend their axiomatic foundation for the integer DEA under VRS, non-increasing, and non-decreasing returns to scale. Khezrimotlagh, et al. [25] critique these two models and show that the input targets obtained from the model proposed by Kuosmanenand Matin [11] and Kazemi Matinand Kuosmanen [24] may not be less than those computed by the model developed by Lozanoand Villa [23]. Jie, et al. [26] provide a technical note on the model proposed by Kuosmanenand Matin [11] and improve their model into a rectified model. They show that the new model can effectively answer the problem of a counter case studied by Khezrimotlagh, et al. [25]. Since additive models target slacks directly in reporting the efficiency, they reveal higher discrimination power especially in the presence of integer values. Du, et al. [12] propose new models based on Andersen and Petersen’s technique [27] in which slacks are directly investigated in order to compute efficiency and super-efficiency scores when inputs and outputs are integer-valued.

For the purpose of incorporating flexible measures, Cookand Zhu [16] present a modification of the standard CCR DEA model and illustrate its application in two practical problem settings. They develop their model using the MILP approach to suggest both a specific DMU model and an aggregate model as methods to originate suitable descriptions for flexible measures. However, their technique may report incorrect inefficiency indices attributable to a computational problem as a result of utilizing a large positive number in their model. This situation is addressed by Toloo [28]. He revises Cook and Zhu’s model so that it does not need to introduce a large positive number. The methodology classifies flexible measures either as input or output according to their contribution to technical efficiency optimization (optimum solution) based on MILP housing both possibilities simultaneously. Afterward, several studies try to propose further refinements [17, 29–31]. Some of the researchers also try to provide an ensuing and instructive discussion on the infeasibility issues of these models such as Amirteimooriand Emrouznejad [32] and Sedighi Hassan Kiyadeh, et al. [33]. The flexible SBM (FSBM) models have recently been addressed by some studies. Amirteimoori, et al. [34] introduce an FSBM for calculating the efficiency score where flexible measures are present. They show that if a DMU is perceived as efficient the flexible measure can play both input and output roles. Tohidiand Matroud [35] develop an alternative non-oriented model to classify the status of flexible measures and determine returns to scale setting.

There are as well situations in the real world where certain measures can play either input or output roles and can only take integer values, for instance, the number of graduates. Such real situations result in new unified DEA models in which both integer-valued amounts and flexible measures are simultaneously addressed. Kordrostami, et al. [36] contribute to this topic by proposing an additive slacks-based approach which is also treatable under both VRS and CRS environments. However, additive models do not directly calculate the efficiency score of the DMU under evaluation in their objective function. Therefore, the final efficiency score can be (post) calculated using the SBM DEA model’s definition. However, as pointed out by Khezrimotlagh, et al. [37] the score of SBM for the additive model may not result in an appropriate efficiency score. The SBM model measures the maximum possible slacks to minimize the efficiency score, whereas the additive model measures the maximum possible slacks without concerning the minimum efficiency score [2]. Therefore, the proposed model by Kordrostami, et al. [36] which is based on Du, et al. [12] may not report all inefficiencies (efficiency scores) correctly. Another issue that can be recognized is the way the flexible measures have been addressed in the FSBM models such as in Amirteimoori, et al. [34]. They address the flexible measures in a way that deviates from the standard SBM. Since flexible measures can simultaneously be designated as input and output in the objective function, the averages in both the numerator (input excess) and denominator (output shortfall) are respectively computed using the fixed numbers of inputs plus the flexible measures and the fixed numbers of outputs plus the flexible measures regardless of the optimum solution where the status of the flexible measure is determined. Consequently, the efficiency score is overestimated compared to the efficiency score obtained from the standard SBM. Furthermore, the flexible measure may be differently classified for some DMUs. Boďa [38] addresses this issue by proposing a modified model that distinguishes the same efficient and inefficient DMUs as Amirteimoori, et al. [34], however, realizes different projections for inefficient DMUs which means different classifications of flexible measures.

This study proposes an SBM model in which both flexible and integer measures are simultaneously presented. The main advantage of the proposed model is all input, output, and flexible measures can take integer-valued quantities without fluctuating the efficiency level. In addition, the mathematical reformulation of the proposed models considers two main properties of the SBM DEA model: units-invariant and monotone decreasing in slacks. Furthermore, the technical efficiency score is directly calculated in the proposed SBM model and inflation of scores is prevented by modifying the input and output inefficiencies. The proposed model is developed based on the MILP approach then, can be easily solved by most non-commercial and open-source solvers. Furthermore, slack values of inputs, outputs, and flexible measures calculated by the proposed model are reported and compared with those obtained from Kordrostami, et al. [36]. However, the same efficient and inefficient DMUs are detected as Kordrostami, et al. [36], the projections for inefficient DMUs and, consequently, classifications of flexible measures are different from each other. We also propose a new objective function for the model developed by Kordrostami, et al. [36] so that the new additive efficiency index falls between zero and one. The applicability of the introduced models is illustrated and scrutinized via a real-case dataset of German university hospitals. The main practical goal is to indicate the magnitude and source of inefficiencies for the university hospitals. This might support both local and national health authorities in decision-making processes including resource allocation, utilization, and planning.

## Slacks-based measure data envelopment analysis

We first present progress made in integer-valued and flexible DEA models in literature before moving on to the final proposed model. By doing this, readers should be able to better understand how the models have evolved over the past two decades. Moreover, it allows us to point out how our proposed model advances other models by investigating their differences and commonalities. To begin with, it is worth mentioning the notations used in this paper as follows:

*Sets and indices*:

- *N*: set of DMUs, *N* = {1, …, *n*}
- *I*: set of real-valued inputs, *I* = {1, …, *m*}
- *O*: set of real-valued outputs, *O* = {1, …, *s*}
- *K*: set of real-valued flexible measures, *K* = {1, …, *p*}
- *I*^*I*^: set of the integer-valued inputs, *I*^*I*^ = {1, …, *m*^*I*^}
- *I*^*NI*^: set of the non-integer valued inputs, *I*^*NI*^ = {1, …, *m*^*NI*^}
- *O*^*I*^: set of the integer-valued outputs, *O*^*I*^ = {1, …, *s*^*I*^}
- *O*^*NI*^: set of the non-integer-valued outputs, *O*^*NI*^ = {1, …, *s*^*NI*^}
- *K*^*I*^: set of integer-valued flexible measures, *K*^*I*^ = {1, …, *p*^*I*^}
- *K*^*NI*^: set of non-integer-valued flexible measures, *K*^*NI*^ = {1, …, *p*^*NI*^}
- *j*: index of DMUs, *j* ∈ *N* = {1, …, *n*}
- *i*: index of inputs *i* ∈ *I* = *I*^*I*^ ∪ *I*^*NI*^
- *r*: index of outputs *r* ∈ *O* = *O*^*I*^ ∪ *O*^*NI*^
- *k*: index of flexible measures *k* ∈ *K* = *K*^*I*^ ∪ *K*^*NI*^

*Parameters*:

- *x*_*ij*_: real-valued amounts of input *i* utilized by *DMU*_*j*_
- *y*_*rj*_: real-valued amounts of output *r* produced by *DMU*_*j*_
- *z*_*kj*_: real-valued amounts of flexible measure *k* utilized/produced by *DMU*_*j*_

*Decision variables*:

- *λ*_*j*_: coefficients of the convex linear combination
- ${s}_i^x$: real-valued amounts of input *i* (excess)
- ${s}_r^y$: real-valued amounts of output *r* (shortfall)
- ${s}_{1k}^z,{s}_{2k}^z$: real-valued amounts of flexible measure *k* slack designated as input and output, respectively
- $\tilde{s}_i^x$: integer-valued amounts of input *i* (excess)
- $\tilde{s}_r^y$: integer-valued amounts of output *r* (shortfall)
- $\tilde{s}_{1k}^z,\tilde{s}_{2k}^z$: integer-valued amounts of flexible measure *k* slack designated as input and output, respectively
- $\tilde{d}_k,{d}_k$: binary variables to indicate the role of integer- and non-integer valued flexible measure *k*, (respectively)

*Auxiliary variables*:

- $\tilde{x}_{ij}$: integer-valued reference point for input *i* utilized by *DMU*_*j*_
- $\tilde{y}_{rj}$: integer-valued reference point for output *r* produced by *DMU*_*j*_
- $\tilde{z}_{kj}$: integer-valued reference point for flexible measure *k* utilized/produced by *DMU*_*j*_
- ${s^{\prime}}_{1k}^z,{s^{\prime}}_{2k}^z$: auxiliary variables for real flexible measure k as input and output, respectively
- ${\delta}_i^x$: auxiliary variable for the real input *i* (excess)
- ${\delta}_r^y$: auxiliary variable for the real output *r* (shortfall)
- ${\delta}_{1k}^z,{\delta}_{2k}^z$: auxiliary variables for real flexible measure *k* as input and output, respectively
- ${\tilde{s}^{\prime}}{}^{z}_{1k},{\tilde{s}^{\prime}}{}^{z}_{2k}$: auxiliary variables for integer flexible measure *k* as input and output, respectively
- ${\overset{\sim }{\delta}}_i^x$: auxiliary variable for the integer input *i* (excess)
- ${\overset{\sim }{\delta}}_r^y$: auxiliary variable for the integer output *r* (shortfall)
- ${\overset{\sim }{\delta}}_{1k}^z,{\overset{\sim }{\delta}}_{2k}^z$: auxiliary variables for integer flexible measure *k* as input and output, respectively
- ${a}_{k^{\prime }},\tilde{a}_{k^{\prime }}$: auxiliary binary decision variables

Now, assume we have *n* DMUs, *DMU*_*j*_ ∀ *j* = 1, …, *n*, that utilize *m* inputs (real-valued inputs), *x*_*ij*_, ∀ *j*, *i* = 1, …, *m* to produce *s* outputs (real-valued outputs) *y*_*rj*_, ∀ *j*, *r* = 1, …, *s*. The inputs and outputs can take only positive values^1^ i.e., ***x***, ***y*** > **0**. Then, the SBM DEA model proposed by Tone [2] can be formulated as:

$$\left[ SBM\right]$$

1.1 $${\uprho}_h^{SBM}=\mathit{\operatorname{Min}}\ \frac{1-{m}^{-1}\left[\sum_{i\in I}\frac{s_i^x}{x_{ih}}\right]}{1+{s}^{-1}\left[\sum_{r\in O}\frac{s_r^y}{y_{rh}}\right]}$$

1.2 $$s.t.\; {x}_{ih}={\sum}_{j=1}^n{\lambda}_j{x}_{ij}+{s}_i^x,\; \forall i\in I$$

1.3 $${y}_{rh}={\sum}_{j=1}^n{\lambda}_j{y}_{rj}-{s}_r^y,\; \forall r\in O$$

1.4 $${\lambda}_j,{s}_i^x,{s}_r^y\ge 0,\; \forall j,i,r$$

where ${\uprho}_h^{SBM}$is the SBM efficiency score of the unit under evaluation *DUM*_*h*_. ***λ =*** (*λ*_1_, …, *λ*_*n*_) is called the intensity vector which identifies the reference sets for *DMU*_*h*_. ${\boldsymbol{s}}^x=\left({s}_1^x,\dots, {s}_m^x\right)$ and ${\boldsymbol{s}}^y=\left({s}_1^y,\dots, {s}_r^y\right)$ are respectively representing the input and output slacks. Note that Model (1) and the following models are formulated under the CRS setting, however, they can be reformulated under the VRS setting by simply adding ${\sum}_{j=1}^n{\lambda}_j=1$ to the set of constraints.

### Integer-valued SBM DEA model

Suppose that some of the inputs and outputs are only valid in integer form. The input set *I* = *I*^*I*^ ∪ *I*^*NI*^, where *I*^*I*^ shows the index of the integer-valued inputs and *I*^*NI*^ shows the index of the rest of the inputs (non-integers). Similarly, the output set *O* = *O*^*I*^ ∪ *O*^*NI*^. To analyze the efficiency score of DMUs in the presence of integer-valued quantities, Model (1) can be straightforwardly formulated based on the PPS defined by Du, et al. [12]. Accordingly, the Integer-valued SBM (ISBM) DEA model can be written as follows:

$$\left[ ISBM\right]$$

2.1 $${\displaystyle \begin{array}{cc}s.t.& {\uprho}_h^{ISBM}=\mathit{\operatorname{Min}}\ \frac{1-{m}^{-1}\left[{\sum}_{\begin{array}{c}i\in {I}^{NI}\end{array}}\frac{s_i^x}{x_{ih}}+{\sum}_{\begin{array}{c}i\in {I}^I\end{array}}\frac{\tilde{s}_i^x}{x_{ih}}\right]}{1+{s}^{-1}\left[{\sum}_{\begin{array}{c}r\in {O}^{NI}\end{array}}\frac{s_r^y}{y_{rh}}+{\sum}_{\begin{array}{c}r\in {O}^I\end{array}}\frac{\tilde{s}_r^y}{y_{rh}}\right]}\end{array}}$$

2.2 $${x}_{ih}={\sum}_{j=1}^n{\lambda}_j{x}_{ij}+{s}_i^x,\; \forall i\in {I}^{NI}$$

2.3 $${y}_{rh}={\sum}_{j=1}^n{\lambda}_j{y}_{rj}-{s}_r^y,\; \forall r\in {O}^{NI}$$

2.4 $$\tilde{x}_{ih}\ge {\sum}_{j=1}^n{\lambda}_j{x}_{ij},\; \forall i\in {I}^I$$

2.5 $$\tilde{x}_{ih}={x}_{ih}-\tilde{s}_i^x,\; \forall i\in {I}^I$$

2.6 $$\tilde{y}_{rh}\le {\sum}_{j=1}^n{\lambda}_j{y}_{rj},\; \forall r\in {O}^I$$

2.7 $$\tilde{y}_{rh}={y}_{rh}-\tilde{s}_r^y,\; \forall r\in {O}^I$$

2.8 $${\lambda}_j,{s}_i^x,{s}_r^y,\tilde{s}_i^x,\tilde{s}_r^y\ge 0,\; \forall j,i,r,k$$

2.9 $$\tilde{x}_{ih},\tilde{y}_{rh}\ integer\forall i\in {I}^I,r\in {O}^I$$

where ${\uprho}_h^{ISBM}$ shows the efficiency score of *DMU*_*h*_ in the presence of integer measures. Du et al. [11] ‘s model does not offer a zero-to-one integrated efficiency score, as in the standard additive DEA model. Model (2) differs from the Du, et al. [12] model in its definition of the efficiency index (the objective function), which mirrors SBM’s efficiency score [2]. Variables $\tilde{s}_i^x$ and $\tilde{s}_r^y$ are respectively non-radial slacks for integer-valued inputs and outputs while variables $\tilde{x}_{ih}\ \textrm{and}\ \tilde{y}_{rh}\in {\mathbb{Z}}^{+}$are the integer-valued reference points (targets) for inputs and outputs of *DMU*_*h*_, respectively. The slack variables $\tilde{s}_i^x$ and $\tilde{s}_r^y$ signify the absolute difference between the reference points ($\tilde{x}_{ih}$ and $\tilde{y}_{rh}$) and the integer-valued inputs and outputs. As shown in Fig. 1, under the VRS setting, the integer DEA targets may not lie within the feasible area (the convex hull). That is why the modeling of the relationship between the convex linear combination and the integer-valued targets i.e., Eqs. (2.4) and (2.5) for integer-valued inputs, and Eqs. (2.6) and (2.7) for integer-valued outputs are slightly different from real-valued counterparts in Model (1). In other words, by defining the integer-valued reference points ($\tilde{x}_{ih}\ \textrm{and}\ \tilde{y}_{rh}$) we guarantee the feasibility of the integer DEA model [12].

### Flexible SBM DEA model

Consider *p* flexible measures shown by *z*_*kj*_, ∀ *j* = {1, …, *n*}, *k* = {1, …, *p*} whose statuses (input or output) are unknown. To incorporate these measures, Model (1) can be reformulated based on the SBM model proposed by Amirteimoori, et al. [34] for classifying the flexible measures as follows:

$$\left[ FSBM\right]$$

3.1 $${\displaystyle \begin{array}{cc}s.t.& {\uprho}_h^{FSBM}=\mathit{\operatorname{Min}}\ \frac{1-{\left(m+\left(p-{\sum}_{k=1}^p{d}_k\right)\right)}^{-1}\left[{\sum}_{i\in I}\frac{s_i^x}{x_{ih}}+{\sum}_{k\in K}\frac{s_{1k}^z}{z_{kh}}\right]}{1+{\left(s+{\sum}_{k=1}^p{d}_k\right)}^{-1}\left[{\sum}_{r\in O}\frac{s_r^y}{y_{rh}}+{\sum}_{k\in K}\frac{s_{2k}^z}{z_{kh}}\right]}\end{array}}$$

3.2 $${x}_{ih}={\sum}_{j=1}^n{\lambda}_j{x}_{ij}+{s}_i^x,\; \forall i\in I$$

3.3 $${y}_{rh}={\sum}_{j=1}^n{\lambda}_j{y}_{rj}-{s}_r^y,\; \forall r\in O$$

3.4 $${z}_{kh}={\sum}_{j=1}^n{\lambda}_j{z}_{kj}+{s}_{1k}^z-{s}_{2k}^z,\; \forall k\in K$$

3.5 $${s}_{1k}^z\cdot {s}_{2k}^z=0,\; \forall k\in K$$

3.6 $${\lambda}_j,{s}_i^x,{s}_r^y,{s}_{1k}^z,{s}_{2k}^z\ge 0,\forall j,i,r,k$$

where ${s}_{1k}^z$ and ${s}_{2k}^z$ are the slacks vectors responding to the flexible measures treating as inputs and outputs, respectively. ${s}_{1k}^z>0$ results in designating *z*_*ko*_ as input and ${s}_{2k}^z>0$ means *z*_*ko*_ plays the role of output in the PPS. Since *z*_*kh*_ must be either designated as input or output, the unique status of it in the PPS is indicated by Eq. (3.5). The nonlinearity of this constraint can be handled by introducing a large positive number $\mathcal{M}$ and a binary decision variable *d*_*k*_, ∀ *k* that assures one and only one of the variables ${s}_{1k}^z$ and ${s}_{2k}^z$ takes positive (non-zero) values simultaneously. Then, Eq. (3.5) can be replaced with the following equivalent linear constraints:

3.5.1 $${s}_{1k}^z\le \mathcal{M}\cdot \left(1-{d}_k\right),\; \forall k\in K$$

3.5.2 $${s}_{2k}^z\le \mathcal{M}\cdot {d}_k,\; \forall k\in K$$

This condition should be reflected in the objection function Eq. (3.1) as well. In other words, if ${s}_{1k}^z>0,\forall k$ (*d*_*k*_ = 0, ∀ *k*) then ${s}_{2k}^z=0$ (*d*_*k*_ = 1, ∀ *k*) and the total number of inputs is (*m* + *p*) in the numerator consequently, the total number of the outputs in the denominator is (*s*). However, this issue is skipped by Amirteimoori, et al. [34], and the number of inputs and outputs they utilize are (*m* + *p*) and (*s* + *p*), respectively. In other words, they consider the number of flexible measures at the same time in both numerator and denominator of the objective function. This results in overestimating the efficiency score since the second term of both numerator and denominator is underestimated. This issue can be solved by redefining the efficiency score as Eq. (3.1). However, the objective function of Model (3) is non-linear. A linear counterpart of Model (3) is proposed by Boďa [38]. He modifies the FSBM model proposed by Amirteimoori, et al. [34] and proposes the following model:

$$\left[ mFSBM\right]$$

4.1 $${\uprho}_h^{mFSBM}=\mathit{\operatorname{Min}}\ \frac{1-\left[{\sum}_{i\in I}\frac{\delta_i^x}{x_{ih}}+{\sum}_{k\in K}\frac{\delta_{1k}^z}{z_{kh}}\right]}{1+\left[{\sum}_{r\in O}\frac{\delta_r^y}{y_{rh}}+{\sum}_{k\in K}\frac{\delta_{2k}^z}{z_{kh}}\right]}$$

4.2 $${\displaystyle \begin{array}{cc}s.t.& {x}_{ih}={\sum}_{j=1}^n{\lambda}_j{x}_{ij}+{s}_i^x,\; \forall i\in I\end{array}}$$

4.3 $${y}_{rh}={\sum}_{j=1}^n{\lambda}_j{y}_{rj}-{s}_r^y,\; \forall r\in O$$

4.4 $${z}_{kh}={\sum}_{j=1}^n{\lambda}_j{z}_{kj}+{s}_{1k}^z-{s}_{2k}^z,\; \forall k\in K$$

4.5.1 $${s}_{1k}^z\le \mathcal{M}\cdot \left(1-{d}_k\right),\; \forall k\in K$$

4.5.2 $${s}_{2k}^z\le \mathcal{M}\cdot {d}_k,\; \forall k\in K$$

4.6 $${\sum}_{k^{\prime }=0}^p{k}^{\prime}\cdot {a}_{k^{\prime }}={\sum}_{k=1}^p{d}_k$$

4.7 $${\sum}_{k^{\prime }=0}^p{a}_{k^{\prime }}=1$$

4.8 $$-\left(1-{a}_{k^{\prime }}\right)\cdot \mathcal{M}+{\delta}_i^x\cdot \left(m+p-{k}^{\prime}\right)\le {s}_i^x\le \left(1-{a}_{k^{\prime }}\right)\cdot \mathcal{M}+{\delta}_i^x\cdot \left(m+p-{k}^{\prime}\right),\; \forall {k}^{\prime },i$$

4.9 $$-\left(1-{a}_{k^{\prime }}\right)\cdot \mathcal{M}+{\delta}_r^y\cdot \left(s+{k}^{\prime}\right)\le {s}_r^y\le \left(1-{a}_{k^{\prime }}\right)\cdot \mathcal{M}+{\delta}_r^y\cdot \left(s+{k}^{\prime}\right),\; \forall {k}^{\prime },r$$

4.10 $$-\left(1-{a}_{k^{\prime }}\right)\cdot \mathcal{M}+{\delta}_{1k}^z\cdot \left(m+p-{k}^{\prime}\right)\le {s}_{1k}^z\le \left(1-{a}_{k^{\prime }}\right)\cdot \mathcal{M}+{\delta}_{1k}^z\cdot \left(m+p-{k}^{\prime}\right),\; \forall {k}^{\prime },k$$

4.11 $$-\left(1-{a}_{k^{\prime }}\right)\cdot \mathcal{M}+{\delta}_{2k}^z\cdot \left(s+{k}^{\prime}\right)\le {s}_{2k}^z\le \left(1-{a}_{k^{\prime }}\right)\cdot \mathcal{M}+{\delta}_{2k}^z\cdot \left(s+{k}^{\prime}\right),\; \forall {k}^{\prime },k$$

4.12 $${\lambda}_j,{s}_i^x,{s}_r^y,{s}_{1k}^z,{s}_{2k}^z,{s^{\prime}}_{1k}^z,{s^{\prime}}_{2k}^z,{\delta}_i^x,{\delta}_r^y,{\delta}_{1k}^z,{\delta}_{2k}^z\ge 0,\; \forall j,i,r,k$$

4.13 $${d}_k,{a}_{k^{\prime }}\in \left\{0,1\right\},\; \forall k,{k}^{\prime }$$

where the optimal solution of the model determines the source of overestimating efficiency scores ${\sum}_{k=1}^K{d}_k$. This issue can be fixed by introducing an auxiliary binary variable ${a}_{k^{\prime }},\forall {k}^{\prime }=\left\{0,\dots, p\right\}$ which controls the optimized number of flexible measures indicated as outputs ${\sum}_{k=1}^p{d}_k$. Constraints (4.6) to (4.13) ensure that the decision variables ${\delta}_i^x={\left(m+\left(p-{\sum}_{k=1}^p{d}_k\right)\right)}^{-1}\cdot {s}_i^x,\forall i$, ${\delta}_r^y={\left(r+{\sum}_{k=1}^p{d}_k\right)}^{-1}\cdot {s}_r^y,\forall r$, ${\delta}_{1k}^z={\left(m+\left(p-{\sum}_{k=1}^p{d}_k\right)\right)}^{-1}\cdot {s}_{1k}^z,\forall k$, and ${\delta}_{2k}^z={\left(s+{\sum}_{k=1}^p{d}_k\right)}^{-1}\cdot {\delta}_{2k}^z,\forall k$. Therefore, the efficiency score is calculated based on the correct total number of inputs and outputs. Eqs. (4.6) and (4.7) ensure the abovementioned equalities are accomplished only and only for ${k}^{\prime }={\sum}_{k=1}^p{d}_k$ (equivalently, ${a}_{\sum_{k=1}^p{d}_k}=1$) in Constraints (4.8) to (4.11) otherwise, they turn into free limits. The conditions defined for flexible measures in Model (3) are valid in this model as well. Let *d*_*k*_ = 1 then ${s}_{1k}^z=0,\forall k$, ${s}_{2k}^z>0,\forall k$, and the flexible measure *z*_*ko*_ is designated as output. In contrast, if *d*_*k*_ = 0 then *z*_*ko*_ plays the role of input.

### Integer-valued flexible SBM DEA model

In the presence of both integer and flexible measures (*K* = *K*^*I*^ ∪ *K*^*NI*^), Kordrostami, et al. [36] develop the additive model proposed by Du, et al. [12] to assess the relative efficiency. Our first step towards assessing the model’s properties is to write the model as follows:

$$\left[ FISBM\right]$$

5.1 $${\uptau}_h^{FISBM}=\mathit{\operatorname{Max}}\ {\sum}_{\begin{array}{c}i\in {I}^{NI}\end{array}}\frac{s_i^x}{x_{ih}}+{\sum}_{\begin{array}{c}i\in {I}^I\end{array}}\frac{\tilde{s}_i^x}{x_{ih}}+{\sum}_{\begin{array}{c}k\in {K}^{NI}\end{array}}\frac{s_{1k}^z}{z_{kh}}+{\sum}_{\begin{array}{c}k\in {K}^I\end{array}}\frac{\tilde{s}_{1k}^z}{z_{kh}}+{\sum}_{\begin{array}{c}r\in {O}^{NI}\end{array}}\frac{s_r^y}{y_{rh}}+{\sum}_{\begin{array}{c}r\in {O}^I\ \end{array}}\frac{\tilde{s}_r^y}{y_{rh}}+{\sum}_{\begin{array}{c}k\in {K}^{NI}\end{array}}\frac{s_{2k}^z}{z_{kh}}+{\sum}_{\begin{array}{c}k\in {K}^I\end{array}}\frac{\tilde{s}_{2k}^z}{z_{kh}}$$

5.2 $${\displaystyle \begin{array}{cc}s.t.& {x}_{ih}={\sum}_{j=1}^n{\lambda}_j{x}_{ij}+{s}_i^x,\; \forall i\in {I}^{NI}\end{array}}$$

5.3 $${y}_{rh}={\sum}_{j=1}^n{\lambda}_j{y}_{rj}-{s}_r^y,\; \forall r\in {O}^{NI}$$

5.4 $${z}_{kh}={\sum}_{j=1}^n{\lambda}_j{z}_{kj}+{s}_{1k}^z-{s}_{2k}^z,\; \forall k\in {K}^{NI}$$

5.5 $${s}_{1k}^z\cdot {s}_{2k}^z=0,\; \forall k\in {K}^{NI}$$

5.6 $$\tilde{x}_{ih}\ge {\sum}_{j=1}^n{\lambda}_j{x}_{ij},\; \forall i\in {I}^I$$

5.7 $$\tilde{x}_{ih}={x}_{ih}-\tilde{s}_i^x,\; \forall i\in {I}^I$$

5.8 $$\tilde{y}_{rh}\le {\sum}_{j=1}^n{\lambda}_j{y}_{rj},\; \forall r\in {O}^I$$

5.9 $$\tilde{y}_{rh}={y}_{rh}-\tilde{s}_r^y,\; \forall r\in {O}^I$$

5.10 $$\tilde{z}_{kh}={\sum}_{j=1}^n{\lambda}_j{z}_{kj}+{\tilde{s}^{\prime}}{}^{z}_{1k}-{\tilde{s}^{\prime}}{} ^{z}_{2k},\; \forall k\in {K}^I$$

5.11 $$\tilde{z}_{kh}={z}_{kj}-\tilde{s}_{1k}^z+\tilde{s}_{2k}^z,\; \forall k\in {K}^I$$

5.12 $${\tilde{s}^{\prime}} {}^z_{1k}\cdot {\tilde{s}^{\prime}} {}^z_{2k}=0,\; \forall k\in {K}^I$$

5.13 $$\tilde{s}_{1k}^z\cdot \tilde{s}_{2k}^z=0,\; \forall k\in {K}^I$$

5.14 $${\tilde{s}^{\prime}} {}_{1k}^z\cdot \tilde{s}_{2k}^z =0,\; \forall k\in {K}^I$$

5.15 $$\tilde{s}_{1k}^z\cdot {\tilde{s}^{\prime}} {}_{2k}^z=0,\; \forall k\in {K}^I$$

5.16 $${\lambda}_j,{s}_i^x,{s}_r^y,{s}_{1k}^z,{s}_{2k}^z,\tilde{s}_i^x,\tilde{s}_r^y,\tilde{s}_{1k}^z,\tilde{s}_{2k}^z\ge 0,\forall j,i,r,k$$

5.17 $$\tilde{x}_{ih},\tilde{y}_{rh},\tilde{z}_{kh}\ integer\forall i\in {I}^I,r\in {O}^I,k\in {K}^I$$

where ${\uptau}_h^{FISBM}$ is the maximum summation of slacks. Similar to Model (2), a new integer decision variable $\tilde{z}_{ko},\forall k$ is introduced which represents integer-valued projection points for flexible measure *k* of *DMU*_*o*_. To calculate the efficiency score, Kordrostami, et al. [36] calculate the optimum value of slacks ${\boldsymbol{s}}^{\ast }=\left({{\boldsymbol{s}}^{\ast}}^x,{{\boldsymbol{s}}^{\ast}}^y,{{\overset{\sim }{\boldsymbol{s}}}^{\ast}}^x,{{\overset{\sim }{\boldsymbol{s}}}^{\ast}}^y,{{\boldsymbol{s}}^{\ast}}_1^z,{{\boldsymbol{s}}^{\ast}}_2^z\right)$ and the determined status of flexible measure ***d***^∗^ obtained from Model (5). Then, they use the SBM’s scalar measure as a posteriori efficiency index based on a set of optimal solution from Model (5) as follows:

6 $${\zeta^{\ast FISBM}}_h=\frac{1-{\left(m+\left(p-{\sum}_{k=1}^p{d}_k^{\ast}\right)\right)}^{-1}\left[{\sum}_{\begin{array}{c}i\in {I}^{NI}\end{array}}\frac{{s^{\ast}}_i^x}{x_{ih}}+{\sum}_{\begin{array}{c}i\in {I}^I\end{array}}\frac{{\tilde{s}^{\ast}} {}_i^x}{x_{ih}}+{\sum}_{\begin{array}{c}k\in {K}^{NI}\end{array}}\frac{{s^{\ast}}_{1k}^z}{z_{kh}}+{\sum}_{\begin{array}{c}k\in {K}^I\end{array}}\frac{{\tilde{s}^{\ast}} {}_{1k}^z}{z_{kh}}\right]}{1+{\left(s+{\sum}_{k=1}^p{d}_k^{\ast}\right)}^{-1}\left[{\sum}_{\begin{array}{c}r\in {O}^{NI}\end{array}}\frac{{s^{\ast}}_r^y}{y_{rh}}+{\sum}_{\begin{array}{c}r\in {O}^I\end{array}}\frac{{\tilde{s}^{\ast}} {}_r^y}{y_{rh}}+{\sum}_{\begin{array}{c}k\in {K}^{NI}\end{array}}\frac{{s^{\ast}}_{2k}^z}{z_{kh}}+{\sum}_{\begin{array}{c}k\in {K}^I\end{array}}\frac{{\tilde{s}^{\ast}} {}_{2k}^z}{z_{\textrm{k}h}}\right]}$$

This model (as an additive model) deals directly with both integer- and real-valued input excesses and output shortfalls. However, it has no ratio efficiency term (scalar measure) per se. Model (5) is able to discriminate inefficient from efficient DMUs by looking for slacks, but it is unable to assess the real degree of inefficiency [3, 37]. Mathematically speaking, $\min \left[\frac{1-{\boldsymbol{s}}^x/m}{1+{\boldsymbol{s}}^y/s}\ \right]\not\equiv \max \left[\frac{{\boldsymbol{s}}^x}{m}+\frac{{\boldsymbol{s}}^y}{s}\right]$. For example, consider *s*^*x*^ + *s*^*y*^ = 0.2 + 0.4 = 0.8 which is greater than *s*^*x*^ + *s*^*y*^ = 0.4 + 0.3 = 0.7 but $\frac{1-{s}^x}{1+{s}^y}=\frac{1-0.2}{1+0.4}=0.5$ is not less than $\frac{1-{s}^x}{1+{s}^y}=\frac{1-0.4}{1+0.3}=0.46$. Therefore, we introduce a modified SBM DEA model (hereafter *mFISBM*) in an attempt to define the efficiency index directly based on the slacks and in the presence of integer and flexible measures as Model (7).

$$\left[ mFISBM\right]$$

7.1 $${\uprho}_h^{mFISBM}=\mathit{\operatorname{Min}}\ \frac{1-\left[{\sum}_{\begin{array}{c}i\in {I}^{NI}\end{array}}\frac{\delta_i^x}{x_{ih}}+{\sum}_{\begin{array}{c}i\in {I}^I\end{array}}\frac{{\overset{\sim }{\delta}}_i^x}{x_{ih}}+{\sum}_{\begin{array}{c}k\in {K}^{NI}\end{array}}\frac{\delta_{1k}^z}{z_{kh}}+{\sum}_{\begin{array}{c}k\in {K}^I\end{array}}\frac{{\overset{\sim }{\delta}}_{1k}^z}{z_{kh}}\right]}{1+\left[{\sum}_{\begin{array}{c}r\in {O}^{NI}\end{array}}\frac{\delta_r^y}{y_{rh}}+{\sum}_{\begin{array}{c}i\in {O}^I\end{array}}\frac{{\overset{\sim }{\delta}}_r^y}{y_{rh}}+{\sum}_{\begin{array}{c}k\in {K}^{NI}\end{array}}\frac{\delta_{2k}^z}{z_{kh}}+{\sum}_{\begin{array}{c}k\in {K}^I\end{array}}\frac{{\overset{\sim }{\delta}}_{2k}^z}{z_{kh}}\right]}$$

$${\displaystyle \begin{array}{cc}s.t.& (5.2)\hbox{--} (5.15)\end{array}}$$

7.2 $${\sum}_{\begin{array}{c}{k}^{\prime }=0\end{array}}^{p^{NI}}{k}^{\prime}\cdot {a}_{k^{\prime }}={\sum}_{k=1}^{p^{NI}}{d}_k$$

7.3 $${\sum}_{\begin{array}{c}{k}^{\prime }=0\end{array}}^{p^{NI}}{a}_{k^{\prime }}=1$$

7.4 $$-\left(1-{a}_{k^{\prime }}\right)\cdot \mathcal{M}+{\delta}_i^x\cdot \left({m}^{NI}+{p}^{NI}-{k}^{\prime}\right)\le {s}_i^x\le \left(1-{a}_{k^{\prime }}\right)\cdot \mathcal{M}+{\delta}_i^x\cdot \left({m}^{NI}+{p}^{NI}-{k}^{\prime}\right),\; \forall i\in {I}^{NI},{k}^{\prime }=0,\dots, {p}^{NI}$$

7.5 $$-\left(1-{a}_{k^{\prime }}\right)\cdot \mathcal{M}+{\delta}_r^y\cdot \left({s}^{NI}+{k}^{\prime}\right)\le {s}_r^y\le \left(1-{a}_{k^{\prime }}\right)\cdot \mathcal{M}+{\delta}_r^y\cdot \left({s}^{NI}+{k}^{\prime}\right),\; \forall r\in {O}^{NI},{k}^{\prime }=0,\dots, {p}^{NI}$$

7.6 $$-\left(1-{a}_{k^{\prime }}\right)\cdot \mathcal{M}+{\delta}_{1k}^z\cdot \left({m}^{NI}+{p}^{NI}-{k}^{\prime}\right)\le {s}_{1k}^z\le \left(1-{a}_{k^{\prime }}\right)\cdot \mathcal{M}+{\delta}_{1k}^z\cdot \left({m}^{NI}+{p}^{NI}-{k}^{\prime}\right),\; \forall k\in {K}^{NI},{k}^{\prime }=0,\dots, {p}^{NI}$$

7.7 $$-\left(1-{a}_{k^{\prime }}\right)\cdot \mathcal{M}+{\delta}_{2k}^z\cdot \left({s}^{NI}+{k}^{\prime}\right)\le {s}_{2k}^z\le \left(1-{a}_{k^{\prime }}\right)\cdot \mathcal{M}+{\delta}_{2k}^z\cdot \left({s}^{NI}+{k}^{\prime}\right),\; \forall k\in {K}^{NI},{k}^{\prime }=0,\dots, {p}^{NI}$$

7.8 $${\sum}_{\begin{array}{c}{k}^{\prime }=0\end{array}}^{p^I}{k}^{\prime}\cdot \tilde{a}_{k^{\prime }}={\sum}_{\begin{array}{c}k=1\end{array}}^{p^I}\tilde{d}_k$$

7.9 $${\sum}_{\begin{array}{c}{k}^{\prime }=0\end{array}}^{p^I}\tilde{a}_{k^{\prime }}=1$$

7.10 $$-\left(1-\tilde{a}_{k^{\prime }}\right)\cdot \mathcal{M}+{\overset{\sim }{\delta}}_i^x\cdot \left({m}^I+{p}^I-{k}^{\prime}\right)\le \tilde{s}_i^x\le \left(1-\tilde{a}_{k^{\prime }}\right)\cdot \mathcal{M}+{\overset{\sim }{\delta}}_i^x\cdot \left({m}^I+{p}^I-{k}^{\prime}\right),\; \forall i\in {I}^I,{k}^{\prime }=0,\dots, {p}^I$$

7.11 $$-\left(1-\tilde{a}_{k^{\prime }}\right)\cdot \mathcal{M}+{\overset{\sim }{\delta}}_r^y\cdot \left({s}^I+{k}^{\prime}\right)\le \tilde{s}_r^y\le \left(1-\tilde{a}_{k^{\prime }}\right)\cdot \mathcal{M}+{\overset{\sim }{\delta}}_r^y\cdot \left({s}^I+{k}^{\prime}\right),\; \forall r\in {O}^I,{k}^{\prime }=0,\dots, {p}^I$$

7.12 $$-\left(1-\tilde{a}_{k^{\prime }}\right)\cdot \mathcal{M}+{\overset{\sim }{\delta}}_{1k}^z\cdot \left({m}^I+{p}^I-{k}^{\prime}\right)\le \tilde{s}_{1k}^z\le \left(1-\tilde{a}_{k^{\prime }}\right)\cdot \mathcal{M}+{\overset{\sim }{\delta}}_{1k}^z\cdot \left({m}^I+{p}^I-{k}^{\prime}\right),\; \forall k\in {K}^I,{k}^{\prime }=0,\dots, {p}^I$$

7.13 $$-\left(1-\tilde{a}_{k^{\prime }}\right)\cdot \mathcal{M}+{\overset{\sim }{\delta}}_{2k}^z\cdot \left({s}^I+{k}^{\prime}\right)\le \tilde{s}_{2k}^z\le \left(1-\tilde{a}_{k^{\prime }}\right)\cdot \mathcal{M}+{\overset{\sim }{\delta}}_{2k}^z\cdot \left({s}^I+{k}^{\prime}\right),\; \forall k\in {K}^I,{k}^{\prime }=0,\dots, {p}^I$$

7.14 $${\lambda}_j,{s}_i^x,{s}_r^y,{s}_{1k}^z,{s}_{2k}^z,{s^{\prime}} {}_{1k}^z,{s^{\prime}} {}_{2k}^z,{\delta}_i^x,{\delta}_r^y,{\delta}_{1k}^z,{\delta}_{2k}^z,\tilde{s}_i^x,\tilde{s}_r^y,\tilde{s}_{1k}^z,\tilde{s}_{2k}^z,{\tilde{s}^{\prime}} {}_{1k}^z,{\tilde{s}^{\prime}} {}_{2k}^z,{\tilde{\delta}}_i^x,{\tilde{\delta}}_r^y,{\tilde{\delta}}_{1k}^z,{\tilde{\delta}}_{2k}^z\ge 0,\forall j,i,r,k$$

7.15 $$\tilde{x}_{ih},\tilde{y}_{rh},\tilde{z}_{kh}\ integer\forall i\in {I}^I,r\in {O}^I,k\in {K}^I$$

7.16 $${d}_k,{a}_{k^{\prime }},\tilde{d}_k,\tilde{a}_{k^{\prime }}\in \left\{0,1\right\},\forall k,{k}^{\prime }$$

where similar to Model (4), the set of decision variables {***δ***^*x*^, ***δ***^*y*^, ***δ***^*z*^}, and their equivalents for integer-valued measures (i.e., $\left\{{\overset{\sim }{\boldsymbol{\delta}}}^x,{\overset{\sim }{\boldsymbol{\delta}}}^y,{\overset{\sim }{\boldsymbol{\delta}}}^z\right\}$) make sure that the efficiency score is calculated based on the correct total number of inputs and outputs in the objective function by setting the boundaries of Constraints (7.4) to (7.7) and Constraints (7.10) to (7.13) via introducing the binary decision variable set $\left\{{d}_k,{a}_{k^{\prime }},\tilde{d}_k,\tilde{a}_{k^{\prime }}\right\}$. The nonlinear Constraints (5.12) to (5.15) can be equivalently reformulated as the following set of linear constraints:

5.12.1 $$\tilde{s}_{1k}^z\le \mathcal{M}\cdot \left(1-\tilde{d}_k\right),\; \forall k\in {K}^I$$

5.13.1 $$\tilde{s}_{2k}^z\le \mathcal{M}\cdot \tilde{d}_k,\; \forall k\in {K}^I$$

5.14.1 $${\tilde{s}^{\prime}} {}_{1k}^z \le \mathcal{M}\cdot \left(1-\tilde{d}_k\right),\; \forall k\in {K}^I$$

5.15.1 $${\tilde{s}^{\prime}} {}_{2k}^z\le \mathcal{M}\cdot \tilde{d}_k,\; \forall k\in {K}^I$$

The modified flexible integer-valued SBM DEA model introduced here has all properties of the SBM DEA model originally developed by Tone [2]. The *mFISBM* is units-invariant, i.e., the value of ${\uprho^{\ast}}_h^{mFISBM}$ (≤1) is autonomous of the units in which the inputs, outputs, and flexible measures are assessed. It can as well be confirmed that ${\uprho}_h^{mFISBM}$ is monotone decreasing in all input excesses, output shortfalls, and flexible slacks. To such an extent, a larger value results larger performance score in the attainment of the efficient frontier/facet. ${\uprho^{\ast}}_h^{mFISBM}=1$ means ${{\boldsymbol{s}}^{\boldsymbol{x}}}^{\ast }=\textbf{0},{{\boldsymbol{s}}^{\boldsymbol{y}}}^{\ast }=\textbf{0},{{\boldsymbol{s}}_{\textbf{1}}^{\boldsymbol{z}}}^{\ast }=\textbf{0},{{\boldsymbol{s}}_{\textbf{2}}^{\boldsymbol{z}}}^{\ast }=\textbf{0},{{\overset{\sim }{\boldsymbol{s}}}^{\boldsymbol{x}}}^{\ast }=\textbf{0},{{\overset{\sim }{\boldsymbol{s}}}^{\boldsymbol{y}}}^{\ast }=\textbf{0},{{\overset{\sim }{\boldsymbol{s}}}_{\textbf{1}}^{\boldsymbol{z}}}^{\ast }=\textbf{0},\textrm{and}\ {{\overset{\sim }{\boldsymbol{s}}}_{\textbf{2}}^{\boldsymbol{z}}}^{\ast }=\textbf{0}$, i.e., no real and integer input excesses, no real and integer output shortfalls, and no real and integer flexible measure slacks in any optimal solution. *DMU*_*h*_ $\left({\boldsymbol{x}}_h,{\boldsymbol{y}}_h,{\boldsymbol{z}}_h,{\overset{\sim }{\boldsymbol{x}}}_h,{\overset{\sim }{\boldsymbol{y}}}_h,{\overset{\sim }{\boldsymbol{z}}}_h\right)$ is inefficient if ${\uprho^{\ast}}_h^{mFISBM}<1$. This condition means we have the following expression for inefficient *DMU*_*h*_ $\left({\boldsymbol{x}}_h,{\boldsymbol{y}}_h,{\boldsymbol{z}}_h,{\overset{\sim }{\boldsymbol{x}}}_h,{\overset{\sim }{\boldsymbol{y}}}_h,{\overset{\sim }{\boldsymbol{z}}}_h\right)$: ***x***_*h*_ = ***Xλ***^∗^ + ***s***^***x∗***^, ***y***_*h*_ = ***Yλ***^∗^ − ***s***^***y∗***^, ${\boldsymbol{z}}_h=\boldsymbol{Y}{\boldsymbol{\lambda}}^{\ast }+{{\boldsymbol{s}}_{\textbf{1}}^{\boldsymbol{z}}}^{\ast}-{{\boldsymbol{s}}_{\textbf{2}}^{\boldsymbol{z}}}^{\ast}$ where ${{\boldsymbol{s}}_{\textbf{1}}^{\boldsymbol{z}}}^{\ast}.{{\boldsymbol{s}}_{\textbf{2}}^{\boldsymbol{z}}}^{\ast}=\textbf{0}$, ${\overset{\sim }{\boldsymbol{x}}}_h=\overset{\sim }{\boldsymbol{X}}{\boldsymbol{\lambda}}^{\ast }+{{\overset{\sim }{\boldsymbol{s}}}^{\boldsymbol{x}}}^{\ast}$, ${\overset{\sim }{\boldsymbol{y}}}_h=\overset{\sim }{\boldsymbol{Y}}{\boldsymbol{\lambda}}^{\ast }-{{\overset{\sim }{\boldsymbol{s}}}^{\boldsymbol{y}}}^{\ast}$, ${\overset{\sim }{\boldsymbol{z}}}_h=\overset{\sim }{\boldsymbol{Y}}{\boldsymbol{\lambda}}^{\ast }+{{\overset{\sim }{\boldsymbol{s}}}_{\textbf{1}}^{\boldsymbol{z}}}^{\ast}-{{\overset{\sim }{\boldsymbol{s}}}_{\textbf{2}}^{\boldsymbol{z}}}^{\ast}$ where ${{\overset{\sim }{\boldsymbol{s}}}_{\textbf{1}}^{\boldsymbol{z}}}^{\ast}.{{\overset{\sim }{\boldsymbol{s}}}_{\textbf{2}}^{\boldsymbol{z}}}^{\ast}=\textbf{0}$. Straightforwardly, *DMU*_*h*_ can become efficient by omitting the slacks i.e., ***x***_*h*_ ← ***x***_*h*_ − ***s***^***x∗***^, ***y***_*h*_ ← ***y***_*h*_ + ***s***^***y∗***^, ${\boldsymbol{z}}_h\leftarrow {\boldsymbol{z}}_h-{{\boldsymbol{s}}_{\textbf{1}}^{\boldsymbol{z}}}^{\ast}+{{\boldsymbol{s}}_{\textbf{2}}^{\boldsymbol{z}}}^{\ast}:{{\boldsymbol{s}}_{\textbf{1}}^{\boldsymbol{z}}}^{\ast}\cdot {{\boldsymbol{s}}_{\textbf{2}}^{\boldsymbol{z}}}^{\ast}=\textbf{0}$, ${\overset{\sim }{\boldsymbol{x}}}_h\leftarrow {\overset{\sim }{\boldsymbol{x}}}_h-{{\overset{\sim }{\boldsymbol{s}}}^{\boldsymbol{x}}}^{\ast}$, ${\overset{\sim }{\boldsymbol{y}}}_h\leftarrow {\overset{\sim }{\boldsymbol{y}}}_h+{{\overset{\sim }{\boldsymbol{s}}}^{\boldsymbol{y}}}^{\ast}$, ${\overset{\sim }{\boldsymbol{z}}}_h\leftarrow {\overset{\sim }{\boldsymbol{z}}}_h-{{\overset{\sim }{\boldsymbol{s}}}_{\textbf{1}}^{\boldsymbol{z}}}^{\ast}+{{\overset{\sim }{\boldsymbol{s}}}_{\textbf{2}}^{\boldsymbol{z}}}^{\ast}:{{\overset{\sim }{\boldsymbol{s}}}_{\textbf{1}}^{\boldsymbol{z}}}^{\ast}\cdot {{\overset{\sim }{\boldsymbol{s}}}_{\textbf{2}}^{\boldsymbol{z}}}^{\ast}=\textbf{0}$. These operations can be called *mFISBM-projections* as the *SBM-projection* in Tone [2].

The set of DMUs with the corresponding ***λ***^∗^ > **0** is called reference-set to *DMU*_*h*_ as the SBM DEA model. Furthermore, a DMU is *FISBM-efficient* if and only if it is *mFISBM-efficient* (see Appendix 1). Similar to the SBM model [2], the formulation of ${\uprho}_h^{mFISBM}$ in Model (7) can be interpreted as the product of input and output inefficiencies or the second term of numerator and denominator, correspondingly. Then, the numerator and denominator evaluate, respectively, the mean reduction rate of inputs and mean expansion rate of outputs considering the optimal role of flexible measures as well. It should be noted that when ${\uprho^{\ast}}_h^{mFISBM}=1$, the status of the real- and integre-valued flexible measures cannot be declared for *DMU*_*o*_. As explained by Boďa [38], this is the case of indefinite and it reports technical efficiency score where no matter what *L*-tuple of {0, 1} is taken for $\left\{\boldsymbol{d},\overset{\sim }{\boldsymbol{d}}\right\}$.

The non-oriented *mFISBM* DEA model can be reformulated as input-oriented (IO) by setting the denominator of Eq. (7.1) to one and excluding Constraints (7.4), (7.6), (7.10), and (7.12) from the system. In the same way, the output-oriented (OO) *mFISBM* DEA model can be written but, in this case, we maximize the denominator and set the numerator to 1, and remove Constraints (7.5), (7.7), (7.11), and (7.13) from the model. The oriented *mFISBM* technical efficiency scores are optimal values ${\uprho^{\ast}}_h^{mFISBM- IO}$and $1/{\uprho^{\ast}}_h^{mFISBM- OO}$ where ${\uprho^{\ast}}_h^{mFISBM- IO} and1/{\uprho^{\ast}}_h^{mFISBM- OO}\ge {\uprho^{\ast}}_h^{mFISBM}$. Then, there is no need for the *Charnes–Cooper* transformation (explicitly, no need for dealing with multiplying the scalar variable *t* > 0 in slacks) since both objective functions are linear and the optimum solutions are directly reported by the models.

When dealing with large case studies, there may be a concern about the size (total number of decision variables and constraints) of Model (7) compared to Model (5). This could be problematic from the perspective of computational complexity. To handle this issue, we propose Model (8) with less size than Model (7) as follows (hereafter revised *FISBM* or *rFISBM*):

$$\left[ rFISBM\right]$$

8.1 $${\Omega}_h^{rFISBM}=\mathit{\operatorname{Min}}\; 1-\frac{\left[\begin{array}{c}{\sum}_{\begin{array}{c}i\in {I}^{NI}\end{array}}\frac{{s^{\ast}}_i^x}{x_{ih}}+{\sum}_{\begin{array}{c}i\in {I}^I\end{array}}\frac{{\tilde{s}^{\ast}} {}_i^x}{x_{ih}}+{\sum}_{\begin{array}{c}k\in {K}^{NI}\end{array}}\frac{{s^{\ast}}_{1k}^z}{z_{kh}}+{\sum}_{\begin{array}{c}k\in {K}^I\end{array}}\frac{{\tilde{s}^{\ast}} {}_{1k}^z}{z_{kh}}+{\sum}_{\begin{array}{c}r\in {O}^{NI}\end{array}}\frac{{s^{\ast}}_r^y}{y_{rh}}+{\sum}_{\begin{array}{c}r\in {O}^I\end{array}}\frac{{\tilde{s}^{\ast}} {}_r^y}{y_{rh}}+{\sum}_{\begin{array}{c}k\in {K}^{NI}\end{array}}\frac{{s^{\ast}}_{2k}^z}{z_{kh}}+{\sum}_{\begin{array}{c}k\in {K}^I\end{array}}\frac{{\tilde{s}^{\ast}} {}_{2k}^z}{z_{kh}}\end{array}\right]}{m+s+p}$$

$${\displaystyle \begin{array}{cc}s.t.& (5.2)\hbox{--} (5.17)\end{array}}.$$

Model (8) is also units-invariant and provides an integrated efficiency index (${\Omega}_h^{rFISBM}$) ranging from 0 to 1 (see Appendix 2). ${\Omega}_h^{rFISBM}$can be also called monotonically decreasing with respect to input, output, and flexible slacks so that a larger value represents a smaller slack ratio then, better performance in reaching the efficient frontier. However, unlike ${\uprho}_h^{mFISBM}$, ${\Omega}_h^{rFISBM}$ cannot be construed as the product of input and output inefficiencies. Therefore, the efficiency index calculated by Model (8) cannot be recommended when the investigation of inefficiency sources is the goal of performance evaluation.

### Decomposition of inefficiency

Using optimal slacks ${\boldsymbol{s}}^{\ast }=\left({{\boldsymbol{s}}^{\ast}}^x,{{\boldsymbol{s}}^{\ast}}^y,{{\overset{\sim }{\boldsymbol{s}}}^{\ast}}^x,{{\overset{\sim }{\boldsymbol{s}}}^{\ast}}^y,{{\boldsymbol{s}}^{\ast}}_1^z,{{\boldsymbol{s}}^{\ast}}_2^z\right)$ and the optimal role of integer- and non-integer valued flexible measures $\left({\overset{\sim }{\boldsymbol{d}}}^{\ast },{\boldsymbol{d}}^{\ast}\right)$obtained from the models, we can decompose the inefficiency scores as input and output inefficiencies. These two terms are informative for identifying the sources of inefficiency and the magnitude of their influence on the efficiency score. In other words, the greater the inefficiency, the lower the efficiency score since the inefficiency value is subtracted from the maximum viable efficiency score (=1).

9 $${\displaystyle \begin{array}{cc}\textrm{Input}\ \textrm{inefficiency}:& \frac{\left[{\sum}_{\begin{array}{c}i\in {I}^{NI}\end{array}}\frac{{s^{\ast}}_i^x}{x_{ih}}+{\sum}_{\begin{array}{c}i\in {I}^I\end{array}}\frac{{\tilde{s}^{\ast}} {}_i^x}{x_{ih}}+{\sum}_{\begin{array}{c}k\in {K}^{NI}\end{array}}\frac{{s^{\ast}}_{1k}^z}{z_{kh}}+{\sum}_{\begin{array}{c}k\in {K}^I\end{array}}\frac{{\tilde{s}^{\ast}} {}_{1\textrm{k}}^z}{z_{kh}}\right]}{m^{NI}+{m}^I+\left({p}^{NI}-{\sum}_{k=1}^{p^{NI}}{d}_k^{\ast}\right)+\left({p}^I-{\sum}_{k=1}^{p^I}\tilde{d}_k^{\ast}\right)}\end{array}}$$

10 $${\displaystyle \begin{array}{cc}\textrm{Output}\ \textrm{inefficiency}:& \frac{\left[{\sum}_{\begin{array}{c}r\in {O}^{NI}\end{array}}\frac{{s^{\ast}}_r^y}{y_{rh}}+{\sum}_{\begin{array}{c}r\in {O}^I\end{array}}\frac{{\tilde{s}^{\ast}} {}_r^y}{y_{rh}}+{\sum}_{\begin{array}{c}k\in {K}^{NI}\end{array}}\frac{{s^{\ast}}_{2k}^z}{z_{kh}}+{\sum}_{\begin{array}{c}k\in {K}^I\end{array}}\frac{{\tilde{s}^{\ast}} {}_{2k}^z}{z_{kh}}\right]}{s^{NI}+{s}^I+{\sum}_{k=1}^{p^{NI}}{d}_k^{\ast }+{\sum}_{k=1}^{p^I}\tilde{d}_k^{\ast }}\end{array}}$$

### Analysis of environmental variables

An environmental factor refers to factors that can influence the efficiency of a teaching hospital but are not traditional variables that can be controlled by the manager. A health care organization’s environment may be affected by a variety of factors, including patient needs, a local economy, the location of its facilities, or institutional constraints related to access to capital. The environment in which hospitals operate may seriously bias conclusions if the environment is not adequately accounted for. There must be appropriate factors identified in advance so as to determine whether or not the conditions of the environment in which a hospital operates affect the relative efficiency scores. Due to the lack of data and difficulty in identifying or delineating catchment areas for university hospitals, we use state-specific values. Five factors identified to model the environment in which the university hospitals operate are:

- Population Density. This factor can influence inputs and outputs and thus efficiency. This might be, for example, due to the fact that the lower population density surrounding hospitals could lead to less staff availability and limited access to education and medical care.
- Mortality Rate. This factor indicates the number of deaths per million population. As a result of this factor, we can speculate that a higher mortality rate may be linked to more hospitalizations or a higher previous illness rate.
- Population over 65 (% of the total population). As a demographic indicator, this figure is suitable for investigation, since a higher proportion of older people might result in a higher probability of illness and lower employment rates.
- Education Index. This factor shows the extent to which a federal state’s education system contributes to economic growth and prosperity. It can be taken from the Education Monitor report published by the *Cologne Institute for Economic Research*. Since it is a comprehensive assessment spectrum, from the number of dropouts to new doctoral graduates, this metric seems to be suitable for examining whether different existing educational structures of the various states can influence the teaching performance of the university hospitals under study.
- Gross Domestic Product per Capita (GDP/Capita). One reason this economic indicator deserves attention is that starting and completing a medical degree program (which usually takes six years in Germany) often depends on the financial circumstances of the aspiring students and their parents. In addition, this indicator is suitable for capturing the financial support provided by the federal state, and thus the teaching resources available across German states.

By applying a two-stage analysis, we first solve DEA by taking into account the inputs, outputs, and flexible variables, then regress the efficiency scores (as the dependent variable) on the environmental variables (Ferrier and Valdmanis 1996). The efficiency scores are often fitted with a censored Tobit regression model since they are bounded on both ends of the distribution (0 ≤ *Efficiency Score* ≤ 1). The environmental factors for each university hospital can be defined as a (1 × r) vector ***E***. The Tobit regression is then projected as ${\hat{\delta}}_j={\boldsymbol{E}}_j\boldsymbol{\beta} +{\varepsilon}_j$. The independent variable (the efficiency score of *DMU*_*j*_ or *Eff*_*j*_) can be given by Eq. (11).

11 $$Ef{f}_j=\left\{\begin{array}{c}\ {\hat{\delta}}_j\kern0.75em if\kern0.75em {\hat{\delta}}_j\le 1\\ {}0\kern0.75em if\kern0.75em {\hat{\delta}}_j>1\end{array}\right.$$

where, ${\hat{\updelta}}_{\textrm{j}}$ is the efficiency score obtained from Model (7) and Model (8). **β** is an (r × 1) vector of coefficients that need to be estimated in the stage associated with each contextual (environmental) variable through the maximum (log-)likelihood estimation approach. *ε*_*j*_ is a truncated normal random variable. Simarand Wilson [39], however, show that using efficiency scores as the dependent variable violates the classical regression assumption that variables are independent and identically distributed, which invalidates standard approaches to inference. In this case, it is unwise to draw firm conclusions from conventional statistical tests. Instead, it may be considered exploratory, which variables appear to be most influential on performance.

## Application: The case of German university hospitals

In this section, we use a dataset of 28 public university hospitals in Germany^2^ in 2017. The data collection was carried out in different research steps including homepages of the hospitals and direct contact (e-mail/telephone inquiries) to the responsible departments and proved to be very cumbersome. For inputs, we consider the number of beds, physicians, and nurses. The number of beds is an integer-valued input measure. However, physicians and nurses are in full-time equivalent (FTE) units, i.e., real values. The number of outpatients and case-mix adjusted discharges for inpatients are designated as integer and real outputs, respectively. However, these two outputs as the major outputs for general hospitals, do not provide teaching function. Therefore, we use the number of medical students as the integer-valued output of the university hospitals. The total number of students enrolled in the university hospital’s medical degree programs is reflected in this factor. Since they are not yet trained to practice medicine alongside physicians at a population level, they cannot work in any specialties. This makes them ineligible to be considered as input (trained staff) for university hospitals. However, the degree to which teaching contributes to the training of highly skilled personnel is also an important component in the academic mission performance of a university hospital. Therefore, the total number of medical students is considered as an output [20]. We also introduce two more flexible measures to represent the teaching function in the efficiency assessment: the number of graduates and third-party funding income. Graduates who have completed their doctorate in medicine (trained) in a university hospital can play the role of either input (an available and qualified resource who can work under the supervision of the faculties or physicians so can affect their productivity) or output (accomplished staff, then a benefit resulting from teaching funding). Third-party funding income can be similarly interpreted in the efficiency evaluation of university hospitals; as input (a form of earnings received) or as output since most research-granting agencies are willing to assign funds to the university hospitals with the supreme impact.

Table 1 represents the data of 28 university hospitals with 3 inputs, 3 outputs, 2 flexible measures as well as 5 environmental factors. In the last four rows of the table, the descriptive statistics are reported. The university hospitals considered in this study have on average 1475 beds which are categorized as the large hospital. They employ more than 25,000 and 34,000 FTE physicians and nurses, respectively. From the output perspective, in total over 2.8 million adjusted inpatient admissions and over 11.4 million outpatient visits occurred. The teaching measures show that about 11 thousand graduates and about 84 thousand medical students in these hospitals where have received over €1.5 billion from the research-granting agencies.

**Table 1:** Data of 28 German university hospitals in 2017

	Input Variables			Output Variables			Flexible Variables		Environmental Factors				
DMU	Beds	Physicians	Nurses	Adjusted inpatients	Outpatients	Students	Third-party funding income (**10**^**3**^ €)	Graduates	Population density	Mortality rate	Population ≥ 65	Education index (Points)	GDP/Capita
1	1517	878.81	1124.81	79,965.62	245,085	2339	38,708	330	525.68	11,317.85	20.70	44.20	36,485
2	3011	1998.50	2618.66	224,328.58	1,537,233	7432	153,400	795	4086.13	9537.82	19.20	41.60	37,553
3	1237	725.71	897.95	76,312.64	342,327	2993	40,524	289	525.68	11,317.85	20.70	44.20	36,485
4	1295	852.98	1304.83	97,594.78	428,046	2699	46,882	315	221.43	11,317.85	20.70	44.20	36,485
5	1303	793.38	1035.13	89,673.48	377,545	3232	31,678	386	525.68	10,089.72	20.00	60.40	44,840
6	1378	823.63	1353.20	113,658.93	517,851	3212	37,249	472	185.35	11,317.85	20.70	44.20	36,485
7	1260	831.50	1035.27	85,042.01	226,331	1885	35,505	240	525.68	10,369.09	20.30	49.70	43,833
8	1297	709.43	936.18	81,876.35	276,610	3416	39,286	562	296.75	9800.55	19.80	57.20	43,535
9	1610	1067.65	1274.21	95,335.71	578,049	3090	76,200	459	309.63	11,647.92	21.40	50.00	35,316
10	1554	845.50	1294.47	89,431.96	214,921	2861	52,169	346	167.72	12,698.76	22.80	48.50	25,445
11	919	436.70	715.07	56,000.14	17,095	1598	21,248	204	69.34	13,979.11	25.00	50.70	26,330
12	984	521.20	762.04	58,401.14	169,302	2110	11,473	199	107.98	9646.37	18.90	56.00	61,529
13	1436	1118.10	1468.10	96,848.74	337,455	3347	79,946	405	2438.65	11,647.92	21.40	50.00	35,316
14	1520	834.93	1314.90	118,360.73	459,719	2581	91,368	328	167.72	9800.55	19.80	57.20	43,535
15	1988	1471.50	1573.59	137,557.42	1,093,862	3398	105,465	478	309.63	13,047.00	24.00	64.50	27,354
16	1396	741.83	1130.74	93,933.26	463,361	2334	27,000	315	132.28	11,317.85	20.70	44.20	36,485
17	1464	790.14	1210.11	101,525.98	329,189	3338	98,513	402	525.68	13,039.12	24.90	69.60	28,663
18	1345	773.03	1042.71	86,053.41	296,937	2758	42,977	381	221.43	11,324.20	20.90	50.20	33,605
19	1662	978.73	1314.89	107,254.52	269,380	3417	45,800	442	205.74	9800.55	19.80	57.20	43,535
20	1352	621.37	757.41	69,898.95	214,535	1483	41,913	211	309.63	10,089.72	20.00	60.40	44,840
21	2050	1182.41	1856.37	155,754.00	834,985	5616	96,770	676	185.35	10,089.72	20.00	60.40	44,840
22	1091	949.94	1071.65	86,508.14	254,462	1715	45,585	496	185.35	11,317.85	20.70	44.20	36,485
23	1457	948.44	1228.14	146,479.82	391,521	2777	47,620	293	525.68	10,089.72	20.00	60.40	44,840
24	833	626.85	898.83	69,979.62	154,657	2010	22,220	268	185.35	11,845.80	22.60	44.40	30,475
25	2196	1156.13	1996.66	160,488.88	302,263	3566	63,900	452	183.31	9800.55	19.80	57.20	43,535
26	1559	850.40	1065.25	98,409.18	383,947	2998	90,400	541	309.63	9800.55	19.80	57.20	43,535
27	1150	741.23	813.20	79,745.76	247,370	2736	55,200	340	309.63	10,089.72	20.00	60.40	44,840
28	1438	862.50	1294.44	98,027.93	489,027	2842	36,388	366	185.35	13,039.12	24.90	69.60	28,663
Sum	41,302.0	25,132.5	34,388.8	2,854,447.7	11,453,065.0	83,783.0	1,575,387.0	10,991.0	13,927.46	309,180.68	589.50	1498.00	1,074,867.00
Average	1475.1	897.6	1228.2	101,944.6	409,038.0	2992.3	56,263.8	392.5	497.41	11,042.17	21.05	53.50	38,388.11
StD	430.6	301.6	408.3	35,664.6	305,287.9	1185.6	31,836.8	138.7	825.01	1250.01	1.75	8.12	7832.42
Min	833.0	436.7	715.1	56,000.1	17,095.0	1483.0	11,473.0	199.0	69.34	9537.82	18.90	41.60	25,445.00
Max	3011.0	1998.5	2618.7	224,328.6	1,537,233.0	7432.0	153,400.0	795.0	4086.13	13,979.11	25.00	69.60	61,529.00

The results of efficiency analysis of the teaching universities obtained from Model (5) [36], and the proposed Models (7) and (8) are respectively reported in Tables 4, 5, and 6 in Appendix 3. All three models are run under the CRS setting and implemented in *IBM ILOG CPLEX Optimization Studio*. As might be expected, they exhibit differences and share properties in common. University hospitals 2, 6, 8, 15, 21, 23, and 27 are characterized by all three models as efficient DMUs with the optimum slacks of zero. As claimed in Theorem 1, Model (7) will characterize a DMU as efficient if and only if Model (5) characterizes it as efficient. To interpret the integrality, we run the relaxed form of Model (7) in which the integrality is relaxed. Then, we examine the result of an inefficient unit, say university hospital #9. The optimum objective value of the integrality-relaxed Model (7) ${\uprho^{\ast}}_9^{relaxed}=0.7799$ obtained with the intensity optimum weights $\left\{{\lambda}_2^{\ast }=0.3189,{\lambda}_5^{\ast }=0.1753,\; {\lambda}_{23}^{\ast }=0.0550\right\}$ and other *λ*^∗^ are equal to zero. This set of optimum weights results in the reference input (number of beds) ${\sum}_{j=1}^{28}{\lambda}_j^{\ast }{x}_{Beds,j}=\textrm{1,269.0274}$ which dominates the integer-valued input target obtained from Model (7), $\tilde{x}_{Beds}=1280$. Model (7) implies that $\tilde{x}_{Beds}=1280$ (or 330 units reduction in beds) is a feasible target where is not outside of the real PPS. However, there exist some situations in which the integer-valued reference input is not feasible. For example, consider university hospital #4. The optimum efficiency score obtained from the integrality-relaxed Model (7), ${\uprho^{\ast}}_4^{relaxed}=0.8201$. This yields with the intensity optimum weights $\left\{{\lambda}_6^{\ast }=0.7795,{\lambda}_{12}^{\ast }=0.0245,{\lambda}_{23}^{\ast }=0.0516\right\}$ (others are equal to zero). This reports the reference input (“Beds”) ${\sum}_{j=1}^{28}{\lambda}_j^{\ast }{x}_{Beds,j}=\textrm{1,173.5570}$ which does not dominate the integer-valued input target $\tilde{x}_{Beds}=1049$. The result is due to the designated status of the flexible measures in the final PPS. For the *DMU*_4_, the real flexible measure “Third-party funding income” is detected as input in the non-integer PPS while it plays the role of output in the integer PPS. Therefore, the PPS may not be comparable in some situations where flexible measures can play different roles. Slacks of the convex PPS (produced by the non-integer DEA) are usually real-valued amounts and the optimal integer input/output slacks reported by the models are not constantly a rounding up or down of the real-valued slacks. As reported in Table 5 in Appendix, for example, *DMU*_4_, the integer slacks of “Beds” $\tilde{s}_{Beds}^{\ast }=146$ which is not equal to the rounded up or down of its convex (non-integer) slack ${s}_{Beds}^{\ast }=121.44$. Incontestably, for university hospital #19, $\tilde{s}_{Beds}^{\ast }=39$ differs expressively from its corresponding non-integer slack ${s}_{Beds}^{\ast }=259.58$.

**Fig. 2:**
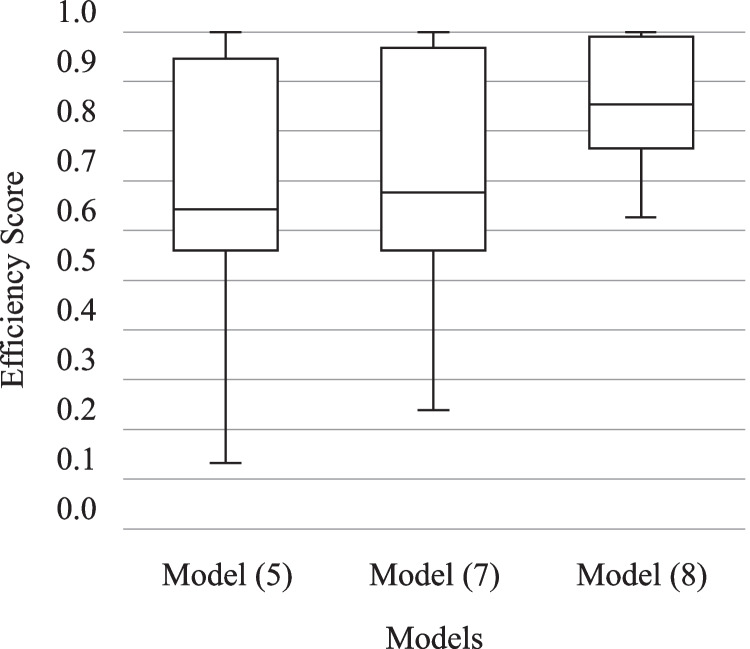
Efficiency scores calculated by Models (5), (7), and (8)

In Tables 4, 5, and 6 in Appendix 3, ${d}_k^{\ast }$ and $\tilde{d}_k^{\ast }$ indicate the roles of “Third-party funding income” and “Graduates” in the final PPS, respectively. In Model (5), 19 out of the 28 university hospitals treat these two flexible measures as output i.e., the majority treats both as output. The same results are reported by Model (7) where 18 and 20 DMUs determine the status of both “Third-party funding income” and “Graduates” as output, correspondingly. However, Model (8) assigns different optimal designations for these two flexible measures so that only 9 and 7 university hospitals identify the role of “Third-party funding income” and “Graduates” respectively as output, i.e., the majority of 19 and 21 DMUs treat them as input. This can explain the difference between the efficiency scores calculated by Models (5) and (7) with those calculated by Model (8) as illustrated in Fig. 2. The inefficiency scores obtained from all three models are asymmetrically distributed since the medians of inefficiency scores are not in the middle of the boxes, and the whiskers are not about the same on the upper and lower sides. These boxes are also advantageous for offering a visual indicator of the variability of inefficiencies. The minimum of 0.6263, the first quartile at 0.7660, and a standard deviation equal to 0.1095, all signify the limited discriminative power of Model (8)‘s inefficiencies in this case. However, the situation changes with Models (5) and (7) where the longer boxes show more dispersed and scattered inefficiency scores. Since the median lines of Model (5) (=0.6434) and Model (7) (=0.6760) are close to each other, there is likely to be no difference between the efficiency scores of these two models. On the other hand, the median line of Model (5) which sits above 0.8534, represents the possibility of a difference between the inefficiency scores calculated by this model and the others. No inefficiency score is detected as the outlier. In other words, the lowest inefficiency score computed by the models is within one and a half interquartile range of its 25th-percentile, and the maximum efficiency score (1.0) is within one and a half of its 75th percentile.

To analyze the magnitudes and sources of inefficiency regarding the corresponding inputs/outputs for each inefficient university hospital, the inefficiency scores can be decomposed using the optimal solution obtained from the models as exhibited in Table 2. This decomposition provides managers or policy-makers with enlightening information about how to become an efficient DMU by examining the magnitudes and sources of inefficiency. For each inefficient university hospital e.g., *DMU*_3_, we can use the optimal slacks obtained from the input-oriented form of Model (7) to calculate input and output inefficiencies via Eqs. (9) and (10), respectively. This indicates the excesses in inputs (*Input Ineffieicncy* = 0.070) dominate shortages in outputs (*Output Ineffieicncy* = 0.0122 ). As might be expected, the majority of the inefficiency sources identified by this model are input inefficiency (17 out of 21) since we run an input-oriented model. This also explains why most of the output slacks and correspondingly output inefficiencies are equal to zero. Those four university hospitals (namely, 5, 12, 16, and 24) in which the output inefficiency is identified as the main source of inefficiency share one significant property in common. They all have a considerable amount of slacks of the flexible measure “Third-party funding income” that is designated as output (${d}_k^{\ast }=1$) in the optimum solutions obtained from all three models (see Tables 4, 5, and 6 in Appendix 3). This indicates the significant shortage in the third-party funding income dominates other inefficiencies. However, this is different for Model (5) where 18 out of 21 inefficiencies are attributed to output inefficiency. In other words, most university hospitals are input efficient. Part of the clarification for the distinct results is that Model (5) is additive and its objective function maximizes the summation of slacks (see Eq. (5.1)) instead of targeting input/output inefficiencies. Therefore, the real degree of inefficiency of the university hospitals can not be assessed.

**Table 2:** Results of the inefficiency decomposition analysis

	Model (5)			Model (7)		
DMU	Input Inefficiency	Output Inefficiency	Dominant	Input Inefficiency	Output Inefficiency	Dominant
1	0.0000	0.6605	Output Inefficiency	0.3092	0.0000	Input Inefficiency
2	0	0	Efficient	0	0	Efficient
3	0.0292	0.1511	Output Inefficiency	0.0700	0.0122	Input Inefficiency
4	0.0752	0.2428	Output Inefficiency	0.1673	0.0021	Input Inefficiency
5	0.0265	0.2363	Output Inefficiency	0.0645	0.1207	Output Inefficiency
6	0	0	Efficient	0	0	Efficient
7	0.0129	0.6903	Output Inefficiency	0.2678	0.0000	Input Inefficiency
8	0	0	Efficient	0	0	Efficient
9	0.2034	0.0318	Input Inefficiency	0.2183	0.0000	Input Inefficiency
10	0.0249	0.6076	Output Inefficiency	0.2410	0.0000	Input Inefficiency
11	0.0881	3.6791	Output Inefficiency	0.1835	0.0348	Input Inefficiency
12	0.0074	1.0592	Output Inefficiency	0.1505	0.4298	Output Inefficiency
13	0.1966	0.2590	Output Inefficiency	0.2813	0.0000	Input Inefficiency
14	0.1704	0.1366	Input Inefficiency	0.1853	0.0000	Input Inefficiency
15	0	0	Efficient	0	0	Efficient
16	0.0203	0.4655	Output Inefficiency	0.1334	0.2007	Output Inefficiency
17	0.1082	0.2251	Output Inefficiency	0.1644	0.0000	Input Inefficiency
18	0.0884	0.3586	Output Inefficiency	0.1705	0.0000	Input Inefficiency
19	0.0636	0.6312	Output Inefficiency	0.1731	0.0000	Input Inefficiency
20	0.1411	0.4423	Output Inefficiency	0.3012	0.0000	Input Inefficiency
21	0	0	Efficient	0	0	Efficient
22	0.2009	0.4104	Output Inefficiency	0.3737	0.0000	Input Inefficiency
23	0	0	Efficient	0	0	Efficient
24	0.1272	0.4113	Output Inefficiency	0.1335	0.1383	Output Inefficiency
25	0.0480	0.4826	Output Inefficiency	0.1682	0.0000	Input Inefficiency
26	0.2039	0.1230	Input Inefficiency	0.2469	0.0000	Input Inefficiency
27	0	0	Efficient	0	0	Efficient
28	0.0128	0.3692	Output Inefficiency	0.1577	0.1149	Input Inefficiency

Now turning to the teaching function, we can see from the reported slacks in Tables 5 and 6 that “Third-party funding income” as one of the teaching proxies has the maximum ratio of slacks (either input excesses or output shortfalls) in all university hospitals except DMU_7_ in Model (7) where excesses in inputs (“Beds”, “Physicians”, and “Nurses”) dominate other inefficiencies. This specifies in almost all the evaluating university hospitals in Germany, teaching inefficiency dominates the general inefficiency. As is now apparent the same result is not seen in the optimum solutions calculated by Model (5) in Table 4 in Appendix 3. In this model, the slacks of “Third-party funding income” are as well substantial while shortages in “Outpatients” are identified as the dominator. This disparity can be due to the fact that Model (5) is additive and its objective function cannot be explained as the inefficiency ratio.

We now investigate the influence of the environmental factors on the efficiency scores, by regressing the efficiency scores obtained from Model (7) and Model (8) against environmental factors, as shown in Table 3. The environmental factors appear to be statistically insignificant, and someone would argue for their exclusion from the DEA analysis on this basis. As a result of the bias in standard errors, the statistical significance may be misestimated - the omission would represent the same as committing a Type I error. Both regression models reveal matching results. In general, the education index of each federal state has a positive impact on the efficiency of university hospitals (but not statistically significant). Assuming a higher education index relates to better academic infrastructure, this can be expected to facilitate attainment and access to education in general. Additionally, a greater proportion of the population over 65 years of age negatively impacts the efficiency scores of university hospitals. It can also be expected that the employment rate will be lower as an older population has less available trained staff and expertise.

**Table 3:** Results of analyzing the environmental variables

Regression Model	Coefficients	Estimate	Std. Error	*z* Value	***Pr***(***>***| ***z***| )	Significant (***α =*** **5**%)
***Model*** _ **7*~Population*** _ ***Density + Mortality*** _ ***Rate + Population ≥*** **65** ***+ Education*** _ ***Index + GDP***	Population Density	0.0001	0.0001	1.2910	0.1970	No
	Mortality Rate	0.0000	0.0002	−0.0560	0.9550	No
	Population ≥ 65	−0.0140	0.1609	−0.0870	0.9310	No
	Education Index	0.0104	0.0118	0.8800	0.3790	No
	GDP/Capita	0.0000	0.0000	−0.2460	0.8050	No
	(Intercept)	0.6322	0.7925	0.7980	0.4250	No
	Log(scale)	−1.3400	0.1654	−8.1020	0.0000	Yes
	Observations	28				
	Log Likelihood	−8.47				
	Akaike Inf. Crit.	30.95				
	Bayesian Inf. Crit.	40.27				
***Model*** _ **8*~Population*** _ ***Density + Mortality*** _ ***Rate + Population ≥*** **65** ***+ Education*** _ ***Index + GDP***	Population Density	0.0000	0.0000	1.1790	0.2385	No
	Mortality Rate	0.0000	0.0001	−0.0890	0.9295	No
	Population ≥ 65	−0.0046	0.0792	−0.0580	0.9540	No
	Education Index	0.0051	0.0058	0.8780	0.3800	No
	GDP/Capita	0.0000	0.0000	−0.5440	0.5863	No
	(Intercept)	0.8620	0.3901	2.2100	0.0271	Yes
	Log(scale)	−2.0490	0.1651	−12.4150	0.0000	Yes
	Observations	28				
	Log Likelihood	6.52				
	Akaike Inf. Crit.	0.97				
	Bayesian Inf. Crit.	10.29				

Despite its small impact, GDP per capita shows a positive impact on the performance of university hospitals. Assumedly, a higher GDP would also allow for more teaching resources to be available. This analysis confirms what we found in the study of inefficiency decomposition, which indicated that third-party funding income can play a principal role in inefficiency sources for German university hospitals.

While German hospitals are credited with reducing hospital and health care expenditure inflation under the Diagnostic Related Group (DRG) based payment system, other significant objectives may be jeopardized by these financial constraints. The university hospital is one type of organization that can be particularly hard hit by budget restrictions. Aside from caring for patients, these hospitals are responsible for training the German medical workforce and conducting medical research. Though both of these endeavors are critical, they are rarely adequately compensated. The results of this study also show how important it is to get more third-party funding incomes (as teaching and research objectives) in order for German university hospitals to become more efficient. In a market where there are few sources of financing, teaching hospitals are in a more competitive position than their non-teaching counterparts. As reported by the German Rectors’ Conference (2016),^3^ to a varying extent, German university hospitals have structural underfunding, which is aggravated by a severe investment lag in building fabric and research infrastructure. Therefore, a substantial and guaranteed increase in their funding allocations is necessary. They aim to establish a well-organized and adequately funded university healthcare system, which does not exist at the moment. Rather, the current framework places hospitals at risk of overshadowing and dominating the research concerns of university medicine due to the economic competition they face. A clear set of regulations should be implemented in order to curtail the cross-subsidization of university hospitals with funds allocated for research and teaching. This means healthcare policymakers need to reconsider the funding structures and strategic plans of German university hospitals and investigate how third-party funding has evolved over time.

It is not easy to evaluate our findings in the light of other studies since there are no recent and comparable studies on the university hospital performance assessment, especially in Germany. However, studies dealing with the efficiency of university hospitals (as discussed in Section 2) have already pointed out differences in efficiency between teaching and non-teaching hospitals. Generally speaking, university hospitals are not able to compete with non-teaching counterparts since they pursue different goals.

## Conclusions

In this study, we advance the SBM DEA model proposed by Tone [2] to consider the real circumstances of the integer nature of certain measures whose status can be flexibly designated. Besides, we develop a revision to the additive model developed by Kordrostami, et al. [36] to make the model report a non-negative inefficiency index with an upper limit of one. Then, the optimal solutions derived from the proposed and revised models are investigated in comparison with Kordrostami’s solutions. This is illustrated by the performance analysis of 28 university hospitals in Germany. In this case study, in addition to the patient care function, the teaching function of the units is captured in the PPS by introducing two flexible measures containing one real-valued (“Third-party funding income”) and one integer-valued (“Graduates”) as well as one integer-valued output (“Students”). In this application, the inclusion of the integrality constraints leads to more valid slacks, i.e., ensures to lie within the integer PPS and not be dominated by any other feasible units. The proposed model describes more reliable and discriminated inefficiency scores from which a more successful ordering of the university hospitals can be originated.

From a practical viewpoint, the decomposition of inefficiencies provides hospital managers, local and national health authorities some informative insights on the source and magnitude of the inefficiency of German university hospitals. The significant shortage in the third-party funding that university hospitals receive as a form of revenue is identified as the main source of inefficiency. Having this fact in mind that most research-granting organizations (e.g., German Research Foundation) consider the university hospitals with the greatest impact, it can be concluded that targeting research missions might boost the efficiency of German university hospitals. Furthermore, hospitals’ operating environments may seriously bias conclusions if they are not adequately taken into account. Therefore, we define five environmental factors in advance to analyze whether the hospital’s operating environment affects its relative efficiency scores via Tobit regression analysis. The results obtained from the second-stage regression analysis confirm that third-party funding income can have a positive impact on the efficiency of German university hospitals. A reconsideration might therefore be required in the university hospital performance management. The enormous public funds that flow into medical education should be allocated more according to efficiency aspects. Now that health care is under increasing pressure to be more efficient due to the introduction of a more results-oriented reimbursement system, similar instruments should also be used for the reimbursement of the academic mission. The proposed SBM DEA model could be used as an accompanying controlling and monitoring instrument. At the same time, in order to avoid cross-subsidies between academic and patient care missions in university hospitals, more transparency is urgently needed by applying a performance assessment approach that allows both missions to be efficiently combined under one roof. Since high-quality teaching cannot be separated from patient care, this realization can give politicians a clear mandate to find a solution to this dilemma. The proposed model could be a suitable monitoring approach for this path, taking into account further comparative parameters and the necessary modifications in the dataset used in the analysis such as identifying new measures.

Different teaching hospitals view their three missions (i.e., patient care, teaching, and research) differently. Some might operationalize these three missions equally. In the application of the proposed DEA model, the role of research is absent due to the lack of data. Furthermore, some variables could be reframed to provide a more “apples-to-apples” comparison. For example, one could look at teaching intensity instead of simply using the number of students. Grosskopf, et al. [19] define teaching intensity as the number of FTE residents per hospital bed. If we look at this measure of teaching intensity regarding the data provided in Table 1, we see considerable variation among the 28 hospitals. Look at DMU’s 20 and 21 for example. If we define teaching intensity as the number of students per bed, DMU 20 has a teaching intensity of 1.1, and DMU 21 has a teaching intensity of 2.7. It would appear that these two hospitals place a much different emphasis on their mission of teaching.

However, the choice of variables (inputs, outputs, and flexible measures) and the measurement of those variables need to be carefully examined in DEA applications otherwise they may present a range of procedural issues. In DEA models, generally, the variables are assumed to be *isotonic*, i.e., an increase in input reduces efficiency while an increase in output increases it [40]. As a result of incorporating ratios into the variable set, a pitfall occurs. It may be acceptable if all variables are of this type, but this can lead to problems when volume measures are mixed in as illustrated by Dyson, et al. [41]. We avoided this pitfall by not including ratios such as teaching intensity into the variable set.

A weakness of the conceptualized model is the lack of quality of patient care in the analysis. However, these datasets are usually classified and are not publicly available. In addition, an attempt should be made to integrate the other university hospitals into the investigation and to conduct an analysis over a longer period of time. A longitudinal study would allow statements on the development of efficiency of individual university hospitals, for instance, in order to assess the efficiency effect of mergers. As a real example, the German Federal Cartel Office^4^ has recently explicated plans to merge the cardiological and cardiosurgical services of the *Charité* and *Deutsches Herzzentrum Berlin* and establish the heart center *Deutsches Herzzentrum der Charité* [42]. Furthermore, from a theoretical perspective, one of the limitations of this study that can be addressed in the future may be extending the present model by incorporating the perspective of the radial characteristics of measure in inefficiency sources. This leads to bringing the effects of inputs/outputs that are subject to change proportionally.

## Data Availability

Not applicable.

## Appendix 1

**Theorem 1**. A DMU is *FISBM-efficient* if and only if it is *mFISBM-efficient*, i.e., ${\uptau^{\ast}}_h^{FISBM}=0\leftrightarrow {\uprho^{\ast}}_h^{mFISBM}=1$.

**Proof**. ${\uptau^{\ast}}_h^{FISBM}=0$ if and only if the optimum value of all inputs, outputs, and flexible slacks be equal to 0 considering the nonnegativity condition imposed by Constraint (5.16), i.e., ${\boldsymbol{s}}^{\ast }=\left({{\boldsymbol{s}}^{\ast}}^x,{{\boldsymbol{s}}^{\ast}}^y,{{\overset{\sim }{\boldsymbol{s}}}^{\ast}}^x,{{\overset{\sim }{\boldsymbol{s}}}^{\ast}}^y,{{\boldsymbol{s}}^{\ast}}_1^z,{{\boldsymbol{s}}^{\ast}}_2^z\right)=\textbf{0}$. By replacing this solution into Model (7), we have ***δ***^*x*^ ≤ **0** and ***δ***^*x*^ ≥ **0** from Constraint (7.8) which results in ***δ***^*x*^ = **0.** The same results about ***δ***^*y*^, ***δ***^*z*^ and ${\overset{\sim }{\boldsymbol{\delta}}}^x,{\overset{\sim }{\boldsymbol{\delta}}}^y,{\overset{\sim }{\boldsymbol{\delta}}}^z$ would be achieved from Constraints (7.4), (7.5), (7.6), (7.10), (7.11), (7.12), and (7.13), respectively. On the other hand, we know that *DMU*_*h*_ in *mFISBM* model is called efficient if and only if ${\uprho^{\ast}}_h^{mFISBM}=1$. This stipulation is equal to ${\boldsymbol{\delta}}^{\ast}=\left({{\boldsymbol{\delta}}^{\ast}}^x,{{\boldsymbol{\delta}}^{\ast}}^y,{{\boldsymbol{\delta}}^{\ast}}^z,{{\overset{\sim }{\boldsymbol{\delta}}}^{\ast}}^x,{{\overset{\sim }{\boldsymbol{\delta}}}^{\ast}}^y,{{\overset{\sim }{\boldsymbol{\delta}}}^{\ast}}^z\right)=\textbf{0}$. This completes the proof. **■**

## Appendix 2

**Theorem 2**. $${\Omega}_o^{rFISBM}\le 1$$.

**Proof**. Assume a solution of *DMU*_*h*_ in which all input excesses (including the flexible measures designated as input) are equal to the corresponding utilized inputs (or their maximum values) i.e., $\left\{{{\boldsymbol{s}}^{\ast}}^x={\boldsymbol{x}}_h,{{\overset{\sim }{\boldsymbol{s}}}^{\ast}}^x={\overset{\sim }{\boldsymbol{x}}}_h,{{\boldsymbol{s}}_1^{\ast}}^z={\boldsymbol{w}}_h,{{{\overset{\sim }{\boldsymbol{s}}}_1}^{\ast}}^z={\overset{\sim }{\boldsymbol{w}}}_h\right\}$. This follows that $$0\le {\left(m+p\right)}^{-1}\left[{\sum}_{\begin{array}{c}i\in {I}^{NI}\end{array}}\frac{{s^{\ast}}_i^x}{x_{ih}}+{\sum}_{\begin{array}{c}i\in {I}^I\end{array}}\frac{{\tilde{s}^{\ast}} {}_i^x}{x_{ih}}+{\sum}_{\begin{array}{c}k\in {K}^{NI}\end{array}}\frac{{s^{\ast}}_{1k}^z}{z_{kh}}+{\sum}_{\begin{array}{c}k\in {K}^I\end{array}}\frac{{\tilde{s}^{\ast}} {}_{1k}^z}{z_{kh}}\right]\le 1$$. However, in the case of outputs, the maximum values for output shortfalls cannot be defined since any output slacks can exceed the corresponding produced outputs $\left\{\textbf{0}\le {{\boldsymbol{s}}^{\ast}}^y,\textbf{0}\le {{\overset{\sim }{\boldsymbol{s}}}^{\ast}}^y,\textbf{0}\le {{\boldsymbol{s}}_2^{\ast}}^z,\textbf{0}\le {{{\overset{\sim }{\boldsymbol{s}}}_2}^{\ast}}^z\right\}$, however, it always holds $$0\le {\left(s+p\right)}^{-1}\left[\begin{array}{c}{\sum}_{\begin{array}{c}r\in {O}^{NI}\end{array}}\frac{{s^{\ast}}_r^y}{y_{rh}}+{\sum}_{\begin{array}{c}r\in {O}^I\end{array}}\frac{{\tilde{s}^{\ast}} {}_r^y}{y_{rh}}+{\sum}_{\begin{array}{c}k\in {K}^{NI}\end{array}}\frac{{s^{\ast}}_{2k}^z}{z_{kh}}+{\sum}_{\begin{array}{c}k\in {K}^I\end{array}}\frac{{\tilde{s}^{\ast}} {}_{2k}^z}{z_{kh}}\end{array}\right]$$. Since the inefficiency scores are non-negative ($0\le {\Omega}_h^{rFISBM}$), this limits the upper bound of the summation of output mix inefficiencies so that the ratio of average input and output inefficiencies cannot take more than 1. It can reach the upper limit, ${\Omega^{\ast}}_h^{rFISBM}=1$, only if slacks are equal to their minimum values defined by Eq. (5.16), i.e., $\left\{{{\boldsymbol{s}}^{\ast}}^x=\textbf{0},{{\boldsymbol{s}}^{\ast}}^y=\textbf{0},{{\overset{\sim }{\boldsymbol{s}}}^{\ast}}^x=\textbf{0},{{\overset{\sim }{\boldsymbol{s}}}^{\ast}}^y=\textbf{0},{{\boldsymbol{s}}^{\ast}}^z=\textbf{0},{{\overset{\sim }{\boldsymbol{s}}}^{\ast}}^z=\textbf{0}\right\}$ which is also a feasible solution for Model (8). **■**

## Appendix 3

**Table 4:** Results of efficiency analysis of university hospitals via Model (5)

DMU	${{\boldsymbol{\uptau}}^{\ast}}_{\boldsymbol{h}}^{\boldsymbol{FISBM}}$	Real Slacks						Integer Slacks						
		Physicians	Nurses	Inpatients	Third-party funding income as input	Third-party funding income as output	${d}_k^{\ast }$	Beds	Outpatients	Students	Graduates as input	Graduates as output	$\tilde{d}_k^{\ast }$	${\upzeta^{\ast}}_h^{FISBM}$
1	3.303	0.00	0.00	21,132.69	0.00	45,754.35	1	0	247,811	843	0	160	1	0.4760
2	0.000	0.00	0.00	0.00	0.00	0.00	1	0	0	0	0	0	0	1.0000
3	0.843	39.52	0.00	2310.42	0.00	5683.70	1	41	0	0	0	169	1	0.7849
4	1.440	105.99	132.17	794.97	0.00	14,250.33	1	0	99,474	848	0	112	1	0.6798
5	1.261	63.19	0.00	214.45	0.00	28,063.17	1	0	27,475	0	0	85	1	0.7061
6	0.000	0.00	0.00	0.00	0.00	0.00	0	0	0	0	0	0	0	1.0000
7	3.490	32.22	0.00	5129.48	0.00	30,963.51	1	0	332,770	1042	0	119	1	0.4614
8	0.000	0.00	0.00	0.00	0.00	0.00	1	0	0	0	0	0	1	1.0000
9	1.112	230.31	176.43	0.00	12,816.52	0.00	0	347	54,914	1	128	0	0	0.6487
10	3.113	0.00	0.00	19,489.45	0.00	16,459.12	1	116	389,319	1024	0	116	1	0.4894
11	18.660	0.00	49.78	0.00	0.00	14,094.23	1	179	296,223	395	0	32	1	0.1328
12	5.318	0.00	0.57	6885.81	0.00	51,051.96	1	21	52,676	0	0	83	1	0.3600
13	1.823	259.89	58.42	21,558.21	41,107.25	0.00	0	0	201,961	1	0	87	1	0.6381
14	1.228	0.00	9.65	0.00	52,655.75	0.00	0	149	23,974	489	0	100	1	0.7299
15	0.000	0.00	0.00	0.00	0.00	0.00	0	0	0	0	0	0	1	1.0000
16	2.388	0.00	0.13	1900.89	0.00	41,436.56	1	85	0	1010	0	107	1	0.5563
17	1.333	0.00	0.00	572.29	37,050.27	0.00	0	83	190,826	421	0	76	1	0.7279
18	1.788	0.00	29.46	744.06	0.00	16,366.99	1	180	297,582	118	73	0	0	0.6710
19	2.779	0.00	32.12	2631.27	0.00	29,331.48	1	187	483,372	224	52	0	0	0.5740
20	2.333	45.75	0.00	0.00	10,492.57	0.00	0	325	11,989	1121	0	202	1	0.5955
21	0.000	0.00	0.00	0.00	0.00	0.00	1	0	0	0	0	0	1	1.0000
22	2.445	228.37	128.18	0.00	0.00	6331.82	1	0	253,996	865	220	0	0	0.5666
23	0.000	0.00	0.00	0.00	0.00	0.00	1	0	0	0	0	0	0	1.0000
24	2.154	113.40	167.11	0.00	0.00	13,546.93	1	0	160,147	0	38	0	0	0.6185
25	2.557	0.00	177.77	0.00	0.00	53,178.41	1	121	387,149	572	0	63	1	0.5383
26	1.388	26.38	0.00	0.00	35,009.23	0.00	0	280	141,659	0	228	0	0	0.6149
27	0.000	0.00	0.00	0.00	0.00	0.00	0	0	0	0	0	0	1	1.0000
28	1.885	33.12	0.10	10,781.51	0.00	31,896.10	1	0	92,511	1067	0	108	1	0.6143

**Table 5:** Results of efficiency analysis of university hospitals via input-oriented Model (7)

DMU	${{\boldsymbol{\uprho}}^{\ast}}_{\boldsymbol{h}}^{\boldsymbol{mFISBM}-\boldsymbol{IO}}$	Real Slacks						Integer Slacks					
		Physicians	Nurses	Inpatients	Third-party funding income as input	Third-party funding income as output	${d}_k^{\ast }$	Beds	Outpatients	Students	Graduates as input	Graduates as output	$\tilde{d}_k^{\ast }$
1	0.3730	278.03	320.53	0.00	0.00	0.00	1	495	0	0	0	0	1
2	1.0000	0.00	0.00	0.00	0.00	0.00	1	0	0	0	0	0	1
3	0.8505	87.93	0.00	817.04	0.00	2034.74	1	110	0	0	0	0	1
4	0.6927	141.97	290.51	0.00	0.00	486.24	1	146	0	0	0	0	1
5	0.8687	98.34	0.46	0.00	0.00	19,115.53	1	90	0	0	0	0	1
6	1.0000	0.00	0.00	0.00	0.00	0.00	1	0	0	0	0	0	1
7	0.4753	242.54	275.07	0.00	0.00	0.00	1	310	0	0	0	0	1
8	1.0000	0.00	0.00	0.00	0.00	0.00	1	0	0	0	0	0	1
9	0.5533	236.00	194.26	0.00	16,860.89	0.00	0	330	0	0	134	0	0
10	0.5021	183.52	301.66	0.00	0.00	0.00	1	424	0	0	0	0	1
11	0.5919	31.62	152.13	0.00	0.00	3699.12	1	244	0	0	0	0	1
12	0.6660	76.90	66.68	0.00	0.00	24,656.44	1	213	0	0	0	0	1
13	0.5785	353.75	434.63	0.00	35,435.49	0.00	0	100	0	0	0	0	1
14	0.6674	0.23	114.65	0.00	47,997.54	0.00	0	195	0	0	0	0	1
15	1.0000	0.00	0.00	0.00	0.00	0.00	1	0	0	0	0	0	1
16	0.7331	2.04	183.74	0.00	0.00	21,677.36	1	284	0	0	52	0	0
17	0.6975	38.12	72.32	0.00	41,829.17	0.00	0	183	0	0	0	0	1
18	0.6648	57.59	73.33	0.00	17,251.91	0.00	0	126	0	0	81	0	0
19	0.6646	86.88	39.81	0.00	21,297.25	0.00	0	39	0	0	114	0	0
20	0.3144	139.27	98.86	0.00	17,769.25	0.00	0	576	0	0	0	0	1
21	1.0000	0.00	0.00	0.00	0.00	0.00	1	0	0	0	0	0	1
22	0.2394	376.22	327.58	0.00	15,517.20	0.00	0	211	0	0	314	0	0
23	1.0000	0.00	0.00	0.00	0.00	0.00	1	0	0	0	0	0	1
24	0.7329	128.27	171.26	0.00	0.00	12,295.51	1	7	0	0	35	0	0
25	0.6339	70.03	436.79	0.00	11,565.67	0.00	0	467	0	0	0	0	1
26	0.4978	76.81	24.63	0.00	52,067.65	0.00	0	299	0	0	191	0	0
27	1.0000	0.00	0.00	0.00	0.00	0.00	1	0	0	0	0	0	1
28	0.6846	107.60	255.90	0.00	0.00	16,717.84	1	247	0	0	50	0	0

**Table 6:** Results of efficiency analysis of university hospitals via Model (8)

DMU	${{\boldsymbol{\Omega}}^{\ast}}_{\boldsymbol{h}}^{\boldsymbol{rFISBM}}$	Real Slacks						Integer Slacks					
		Physicians	Nurses	Inpatients	Third-party funding income as input	Third-party funding income as output	${d}_k^{\ast}$	Beds	Outpatients	Students	Graduates as input	Graduates as output	$\tilde{d}_k^{\ast}$
1	0.7043	241.79	233.92	0.00	16,521.33	0.00	0	403	0	0	100	0	0
2	1.0000	0.00	0.00	0.00	0.00	0.00	0	0	0	0	0	0	0
3	0.9580	87.93	0.00	817.04	0.00	2034.74	1	110	0	0	0	0	1
4	0.8561	149.01	168.98	0.00	15,054.90	0.00	0	122	0	0	0	0	1
5	0.9613	98.34	0.46	0.00	0.00	19,115.53	1	90	0	0	0	0	1
6	1.0000	0.00	0.00	0.00	0.00	0.00	0	0	0	0	0	0	0
7	0.7506	241.97	248.51	0.00	9865.38	0.00	0	305	0	0	47	0	0
8	1.0000	0.00	0.00	0.00	0.00	0.00	1	0	0	0	0	0	0
9	0.7800	239.28	190.01	0.00	18,973.93	0.00	0	341	0	0	122	0	0
10	0.7528	100.17	228.10	0.00	31,822.90	0.00	0	196	0	0	71	0	0
11	0.8337	0.00	101.41	0.00	6322.95	0.00	0	153	0	0	46	0	0
12	0.9097	76.90	66.68	0.00	0.00	24,656.44	1	213	0	0	0	0	1
13	0.7614	279.35	333.13	0.00	50,563.19	0.00	0	0	0	0	34	0	0
14	0.8507	9.80	54.59	0.00	52,680.10	0.00	0	177	0	0	0	72	1
15	1.0000	0.00	0.00	0.00	0.00	0.00	0	0	0	0	0	0	0
16	0.8932	2.04	183.74	0.00	0.00	21,677.36	1	284	0	0	52	0	0
17	0.8490	0.22	161.30	0.00	54,756.14	0.00	0	96	0	0	0	71	1
18	0.8295	57.59	73.33	0.00	17,251.91	0.00	0	126	0	0	81	0	0
19	0.8269	86.88	39.81	0.00	21,297.25	0.00	0	39	0	0	114	0	0
20	0.6933	143.81	133.31	0.00	17,390.89	0.00	0	609	0	0	55	0	0
21	1.0000	0.00	0.00	0.00	0.00	0.00	1	0	0	0	0	0	1
22	0.6263	375.95	327.28	0.00	15,587.54	0.00	0	210	0	0	314	0	0
23	1.0000	0.00	0.00	0.00	0.00	0.00	1	0	0	0	0	0	0
24	0.8932	128.27	171.26	0.00	0.00	12,295.51	1	7	0	0	35	0	0
25	0.8184	42.31	508.89	0.00	15,647.05	0.00	0	389	0	0	88	0	0
26	0.7531	76.81	24.63	0.00	52,067.65	0.00	0	299	0	0	191	0	0
27	1.0000	0.00	0.00	0.00	0.00	0.00	0	0	0	0	0	0	0
28	0.8738	107.60	255.90	0.00	0.00	16,717.84	1	247	0	0	50	0	0

## Footnotes

1 A difficulty arises with zero value measures since the slacks-based ratios are then divided by zero. To handle this problem Tone [2] provides some insights on how to deal with zeros.

2 There exist 35 German university hospitals together with their medical faculties. However, due to the lack of availability of data for seven units, they have been excluded from the analysis. A complete list of German university hospitals is available at https://www.uniklinika.de/.

3 Source: https://www.hrk.de/resolutions-publications/resolutions/beschluss/detail/university-medicine-as-an-integral-part-of-the-university/ (Accessed 01-Aug-2022)

4 In German: *Bundeskartellamt*
